# Activation of SNr GABA neurons drives liver-brain-eye axis dysfunction in hepatic encephalopathy

**DOI:** 10.1016/j.isci.2026.114720

**Published:** 2026-01-16

**Authors:** Kenan Li, Zhenhua Wang, Shaoheng Li, Feifei Wu, Yunhu Bai, Shujiao Li, Changlei Zhu, Ziwei Ni, Shuai Zhang, Yousheng Wu, Fei Tian, Nannan Liu, Tao Chen, Cailian Ruan, Zuoming Zhang, Yanling Yang, Yayun Wang

**Affiliations:** 1 National Demonstrational Center for Preclinical Experimental Medicine, The Fourth Military Medical University, Xi’an, China; 2 Department of Gastrointestinal Surgery, 967 Hospital, Joint Logistics Support Force, PLA, Dalian, China; 3 Department of Hepatobiliary Surgery, Xi-Jing Hospital, The Fourth Military Medical University, Xi’an, China; 4 Department of Ophthalmology, First Affiliated Hospital, The Fourth Military Medical University, Xi’an, China; 5 Aerospace Clinical Medicine Center, The Fourth Military Medical University, Xi’an, China; 6 General Surgery, 988 Hospital, Joint Logistics Support Force, PLA, Zhengzhou, China; 7 Department of Neurobiology, Yan’an University School of Medicine, Yan’an, China

**Keywords:** Molecular physiology, Molecular neuroscience, Neuroanatomy

## Abstract

Hepatic encephalopathy (HE) is frequently accompanied by visual dysfunction, yet the mechanisms underlying the liver-brain-eye axis remain unclear. We established mouse models of acute hepatic encephalopathy (AHE) using thioacetamide and chronic hepatic encephalopathy (CHE) using bile duct ligation, confirming hyperammonemia and visual impairment by electroretinogram (ERG) and visual evoked potentials (VEPs). Retinal analyses revealed preserved structure in AHE, whereas CHE induced significant thinning of the ganglion cell layer (GCL), inner nuclear layer (INL), and outer plexiform layer (OPL). Viral anterograde tracing identified aberrant activation of medial substantia nigra pars reticulata glutamate decarboxylase 2-positive (mSNr^GAD2^) projections to the superior colliculus (SC) under AHE conditions. Chemogenetic inhibition of this pathway restored retinal function, normalized visual signal transmission, and reduced retinal γ-aminobutyric acid (GABA) release. These findings identify SNr-SC signaling as a key neural mechanism driving liver-brain-eye axis dysfunction in HE.

## Highlights

• Viral anterograde tracing with TRAP and GAD2-Cre mice showed SNr activation in AHE
• Chemogenetic inhibition of the mSNr^GAD2^-SC pathway alleviated visual dysfunction
• Aberrant mSNr^GAD2^-SC activation underlies the liver-brain-eye axis in HE

## Graphical abstract

**Figure:**
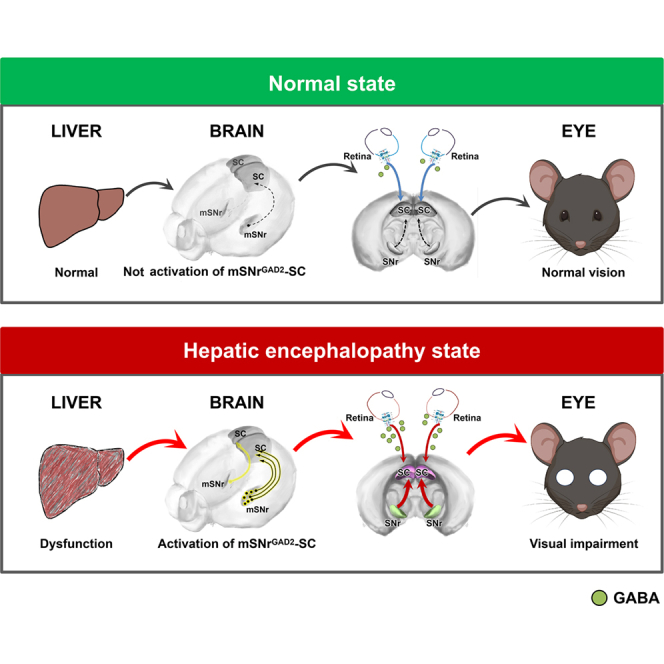

## Introduction

Hepatic encephalopathy (HE) encompasses a spectrum of brain dysfunctions caused by liver insufficiency and/or portosystemic shunting.^1^^,^^2^^,^^3^^,^^4^ Along with ascites and esophageal variceal bleeding, HE is among the most severe complications of decompensated cirrhosis.

Clinical evidence indicates that HE frequently leads to ocular dysfunction. In 1979, Naparstek Y et al.^5^ introduced the term “hepatic cortical blindness” to describe visual cortex damage caused by liver disease, and in 1995, Reichenbach A et al.^6^ proposed the concept of “hepatic retinopathy.” Subsequent studies demonstrated that HE causes a range of visual impairments, including color vision deficits,^7^ abnormal binocular critical flicker frequency,^8^ gaze fixation disturbances,^9^^,^^10^ retinal pathologies,^11^^,^^12^^,^^13^^,^^14^ and optic neuropathy.^15^ In severe cases, these impairments may progress to cortical blindness,^16^^,^^17^ commonly observed in patients with cirrhosis, liver failure, or after liver transplantation. Common triggers include high-protein diets, infections, diuretic use, and electrolyte disturbances. Visual disturbances caused by HE are typically acute, with durations ranging from a few minutes to a month. Recently, researchers have explored automated gaze-tracking systems incorporating machine learning^18^ and visual saccade detection systems^19^ as tools for diagnosing minimal hepatic encephalopathy (MHE). These findings highlight that HE induces a “liver-brain-eye” triad of damage.

The pathogenesis of HE is complex, involving multiple factors such as hyperammonemia, inflammation, oxidative stress, and others. Among these, the ammonia hypothesis remains the dominant explanation for HE pathogenesis.^20^^,^^21^^,^^22^ The substantia nigra (SN) pars reticulata (SNr), a crucial component of the basal ganglia, has been implicated in HE. Consistent clinical imaging findings reveal hyperintense signals in the basal ganglia, especially within the SN, on T1-weighted magnetic resonance imaging (MRI) scans of HE patients,^23^^,^^24^^,^^25^^,^^26^^,^^27^ suggesting that damage to the SN may be a key feature of HE pathophysiology.

Next, we conducted a screening to identify potential neural pathways associated with visual impairment. In our previous study, we found that the SNr specifically responds to elevated brain ammonia levels, with three lines of evidence: first, in the thioacetamide (TAA)-induced acute hepatic encephalopathy (AHE) model, Targeted Recombination in Active Populations (FOS) staining demonstrated selective activation of GABAergic neurons in the SNr, predominantly medial glutamate decarboxylase 2 (GAD2)-positive neurons^28^^,^^29^; second, local intranigral injection of ammonium chloride to simulate high-ammonia conditions within the SNr also enhanced FOS expression in SNr GABAergic neurons^30^; and third, chemogenetic inhibition of medial GAD2-positive neurons in the SNr effectively alleviated motor deficits in HE mice, whereas chemogenetic activation of the same neuronal population in normal mice induced HE-like motor impairments.^31^^,^^32^ Based on these findings, in the present study, we used the SNr as the seed region to trace its projections to downstream nuclei under both normal and AHE conditions.

This research is to utilize TAA and bile duct ligation (BDL) surgery to establish AHE and chronic hepatic encephalopathy (CHE) to showthat abnormal activation of the medial SNr GAD2-positive GABAergic projections to the superior colliculus (SC) (mSNr^GAD2^-SC) pathway serves as the structural basis for the “liver-brain-eye” triad observed in HE. These findings propose a novel therapeutic target for managing visual and systemic complications in liver disease patients.

## Results

### TAA and BDL simulated clinical AHE and CHE conditions, respectively

**Figure 1:**
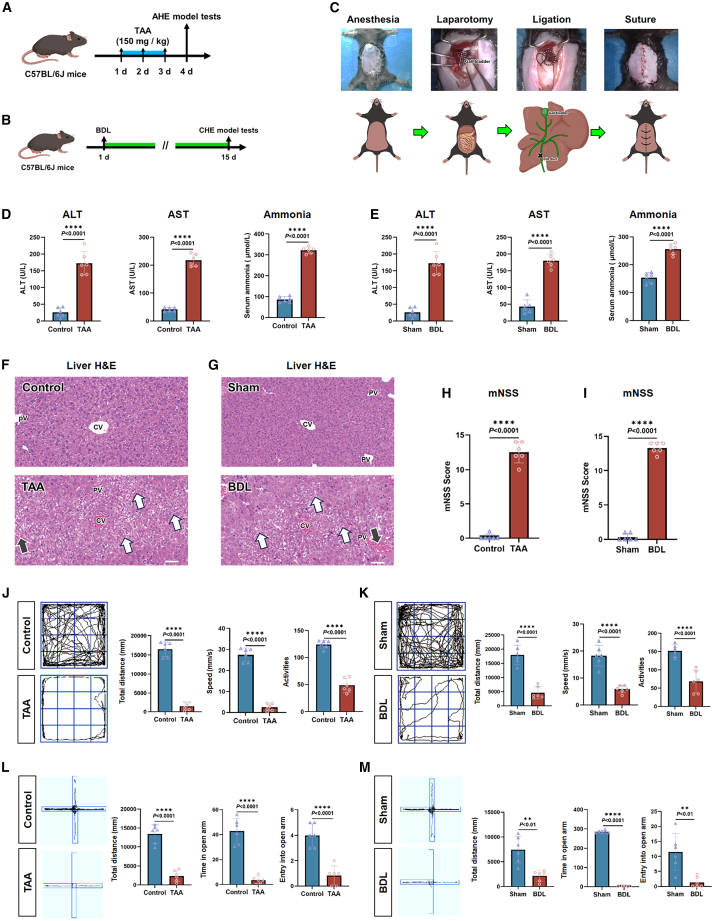
Simulation of AHE and CHE models via TAA intraperitoneal injection and BDL surgery (A) C57BL/6J mice received intraperitoneal injections of TAA (150 mg/kg) for 3 consecutive days to simulate clinical AHE on day 4. (B) C57BL/6J mice underwent BDL surgery on day 1 to simulate clinical CHE on day 15. (C) Key steps in BDL model preparation: anesthesia, laparotomy, isolation and ligation of the bile duct, closure of the abdominal cavity, and suturing of the skin. (D) ELISA assays were performed to measure serum ALT, AST, and ammonia levels in control and AHE mice on day 4 after TAA injection (*n* = 6 per group). (E) ELISA assays were performed to measure serum ALT, AST, and ammonia levels in sham and CHE mice on day 15 after BDL (*n* = 6 per group). (F) Diagram showing H&E staining of liver; tissues from control and AHE mice were collected on day 4 after TAA injection for H&E staining. In the control group, hepatocytes display eosinophilic cytoplasm, large round nuclei centrally located and stained blue, with abundant chromatin and distinct nuclear membranes. Hepatocytes are radially arranged around an intact central vein. In contrast, livers from TAA-treated mice exhibit extensive hepatocyte necrosis and disintegration, accompanied by disruption of the hepatic cord structure (indicated by white arrows), as well as marked sinusoidal dilation, congestion, hemorrhage (indicated by black arrows), and inflammatory cell infiltration in both lobular and portal vein regions; scale bar: 100 μm (top) and 30 μm (bottom). (G) Representative liver H&E staining in sham and CHE groups. The meanings of the black and white arrows are the same as in Figure 1F; scale bar: 100 μm (top) and 30 μm (bottom). (H) mNSS in control and AHE groups (*n* = 6 per group). (I) mNSS in sham and CHE groups (*n* = 6 per group). (J) OFT results in control and AHE groups (*n* = 6 per group). (K) OFT results in sham and BDL groups (*n* = 6 per group). (L) EPM results in control and AHE groups (*n* = 6 per group). (M) EPM results in sham and BDL groups (*n* = 6 per group). All data are presented as mean ± standard deviation. ∗∗*p* < 0.01, ∗∗∗∗*p* < 0.0001 vs. sham. Two-tailed, unpaired, Student’s *t* test for all data.

This study established AHE and CHE^28^^,^^29^^,^^30^^,^^31^^,^^32^ mice. TAA established the AHE model (Figure 1A), and BDL surgery established the CHE model (Figure 1B). Notably, the key steps of the BDL procedure (Figure 1C) included exposing the abdomen, isolating the bile duct, ligating the bile duct, and closing the abdomen. Figure 1D showed that, compared to the control group, serum alanine aminotransferase (ALT) and aspartate aminotransferase (AST) and blood ammonia levels were significantly increased in the TAA group: ALT was elevated by 2-fold (*p* < 0.0001), AST by 4-fold (*p* < 0.0001), and blood ammonia by 2.7-fold (*p* < 0.0001). Further supplementary experiments were conducted to measure the ammonia concentration in the SNr and SC regions of the brain in TAA model mice. The results indicated a significant increase in ammonia levels within these regions (Figure S1). BDL resulted in a 5.7-fold increase in ALT (*p* < 0.0001), a 3-fold increase in AST (*p* < 0.0001), and a 67% increase in blood ammonia (*p* < 0.0001) (Figure 1E). Histological analysis revealed hepatocellular necrosis and disintegration in AHE and CHE models (Figures 1F and 1G). Modified neurological severity score (mNSS) testing revealed neurological impairments in both models (Figures 1H and 1I). Open field tests (OFTs) and elevated plus maze tests (EPMs) indicated significant locomotor dysfunction in both models (Figures 1J–1M). Specifically, the reduced total distance traveled in the OFT reflects general hypoactivity and decreased exploratory drive, while the decreased time spent in the open arms of the EPM indicates heightened anxiety-like behavior—both of which are hallmark neuropsychiatric deficits associated with HE.

### Electrophysiological evidence of visual impairments in AHE and CHE models

**Figure 2:**
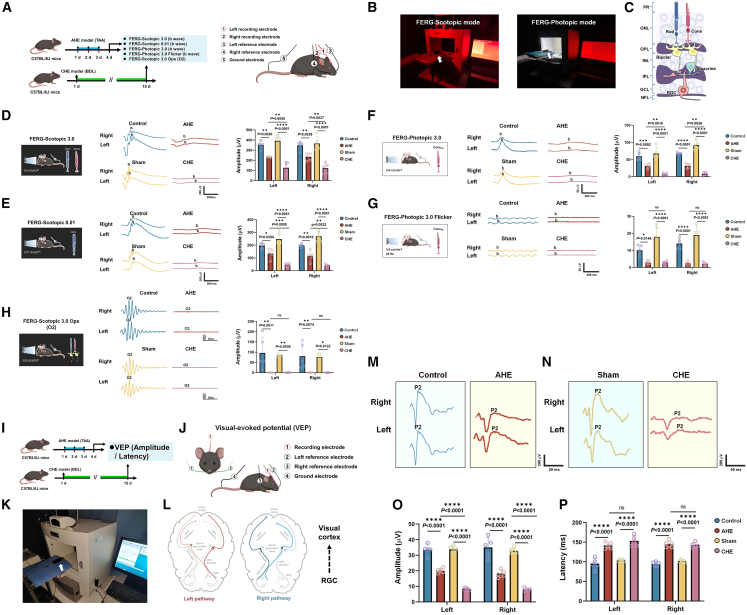
Electrophysiological manifestations of visual impairments in AHE and CHE mice (A) FERG testing was conducted on day 4 in the TAA (AHE) model and day 15 in the BDL (CHE) model; diagram showing recording electrode, reference electrode, and ground electrode positions during FERG testing. (B) Photographs of the dark-adapted (left) and light-adapted (right) testing environments. (C) Schematic of mouse retinal structure. (D) Representative FERG-Scotopic 3.0 waveforms for control and AHE groups (both eyes) and representative FERG-Scotopic 3.0 waveforms for sham and CHE groups (both eyes) (*n* = 5 per group). (E) Representative FERG-Scotopic 0.01 waveforms for control and AHE groups (both eyes) and representative FERG-Scotopic 0.01 waveforms for sham and CHE groups (both eyes) (*n* = 5 per group). (F) Representative FERG-Photopic 3.0 waveforms for control and AHE groups (both eyes) and representative FERG-Photopic 3.0 waveforms for sham and CHE groups (both eyes) (*n* = 5 per group). (G) Representative FERG-Photopic 3.0 Flicker waveforms for control and AHE groups (both eyes) and representative FERG-Photopic 3.0 Flicker waveforms for sham and CHE groups (both eyes) (*n* = 5 per group). (H) Representative FERG-Scotopic 3.0 Ops waveforms for control and AHE groups (both eyes) and representative FERG-Scotopic 3.0 Ops waveforms for sham and CHE groups (both eyes) (*n* = 5 per group). (I) VEP testing was conducted on day 4 in the TAA (AHE) model and day 15 in the BDL (CHE) model. (J) Diagram showing the placement of recording, reference, and ground electrodes. (K) Photograph of the standard VEP testing environment. (L) VEP testing reflects the conduction of visual pathways from the retinal ganglion cells to the visual cortex. (M) Representative VEP waveforms for control and AHE groups (both eyes). (N) Representative VEP waveforms for sham and CHE groups (both eyes). (O) Statistical results of P2 amplitude in VEP testing for control, AHE, sham, and CHE groups (both eyes) (*n* = 5 per group). (P) Statistical results of P2 latency in VEP testing for control, AHE, sham, and CHE groups (both eyes) (*n* = 5 per group). All data are presented as mean ± standard deviation. ∗*p* < 0.05, ∗∗*p* < 0.01, ∗∗∗*p* < 0.001, ∗∗∗∗*p* < 0.0001; ns, not significant vs. sham. Two-tailed, unpaired, Student’s *t* test for all data.

Our team has previously conducted a series of animal studies using visual electrophysiological methods, including electroretinogram (ERG) and visual evoked potentials (VEP).^33^^,^^34^^,^^35^^,^^36^^,^^37^ Figure 2A shows the experimental procedures and electrode connections for Flash Electroretinography (FERG) -Scotopic and FERG-Photopic analyses. Figures 2B and 2C depict retinal cell layers and mouse placement for dark- and light-adapted modes. FERG-Scotopic 3.0 (Figure 2D) showed significant reductions in b-wave amplitude, indicating rod and cone dysfunction in both AHE (33.88% left [*p* < 0.01] and 32.35% right [*p* < 0.01]) and CHE (68.10% left [*p* < 0.0001] and 66.76% right [*p* < 0.0001]) groups, with more severe dysfunction in CHE mice. FERG-Scotopic 0.01 (Figure 2E) revealed significant reductions in rod-specific b-wave amplitude, with AHE showing a 30.68% reduction in the left (*p* < 0.05) and 40.99% in the right (*p* < 0.01) and CHE showing 81.77% (*p* < 0.0001) and 85.14% (*p* < 0.0001) reductions, respectively. FERG-Photopic 3.0 (Figure 2F) indicated central retinal cone dysfunction, with AHE showing reductions of 48.30% in the left (*p* < 0.001) and 53.34% in the right (*p* < 0.0001), while CHE showed more severe reductions (90.46% left [*p* < 0.0001] and 90.95% right [*p* < 0.0001]). FERG-Photopic 3.0 Flicker (Figure 2G) revealed peripheral retinal cone dysfunction, with AHE showing reductions of 71.06% in the left (*p* < 0.05) and 82.83% in the right (*p* < 0.0001), and CHE showing even more severe reductions (84.36% left [*p* < 0.0001] and 88.41% right [*p* < 0.0001]). Neural transmission assessment (Figure 2H) showed significant reductions in both AHE (84.07% left [*p* < 0.01] and 87.30% right [*p* < 0.01]) and CHE (96.65% left [*p* < 0.01] and 96.64% right [*p* < 0.05]) groups.

Using VEP,^35^^,^^36^ we evaluated visual pathway function by recording cortical electrical responses to light stimulation. Testing was conducted on day 4 (AHE) and day 15 (CHE) (Figure 2I). Four electrode placements are shown in Figure 2J, and the testing environment is depicted in Figure 2K. The complete visual pathway, from retinal photoreceptors to the visual cortex, involves multiple neuronal layers, including the optic nerve, optic chiasm, lateral geniculate nucleus, and optic tract (Figure 2L). We measured reductions in P2 wave amplitude and delays in latency to assess transmission abnormalities. Figure 2M presents the VEP results of the left and right eyes for the normal control group and the AHE group. Figure 2N presents the VEP results of the left and right eyes for the sham group and the CHE group. P2 wave amplitude reductions are shown in Figure 2O. In AHE, left and right eye amplitudes decreased by 39.98% (*p* < 0.0001) and 48.23% (*p* < 0.0001), respectively. In CHE, left and right eye amplitudes decreased by 75.44% (*p* < 0.0001) and 75.56% (*p* < 0.0001), respectively. Latency delays are shown in Figure 2P. In AHE, left and right eye latency increased by 36.49% (*p* < 0.0001) and 42.89% (*p* < 0.0001), respectively. In CHE, left and right eye latency increased by 61.39% (*p* < 0.0001) and 50.63% (*p* < 0.0001), respectively.

### Histological and OCT/OCTA analysis in AHE and CHE models

**Figure 3:**
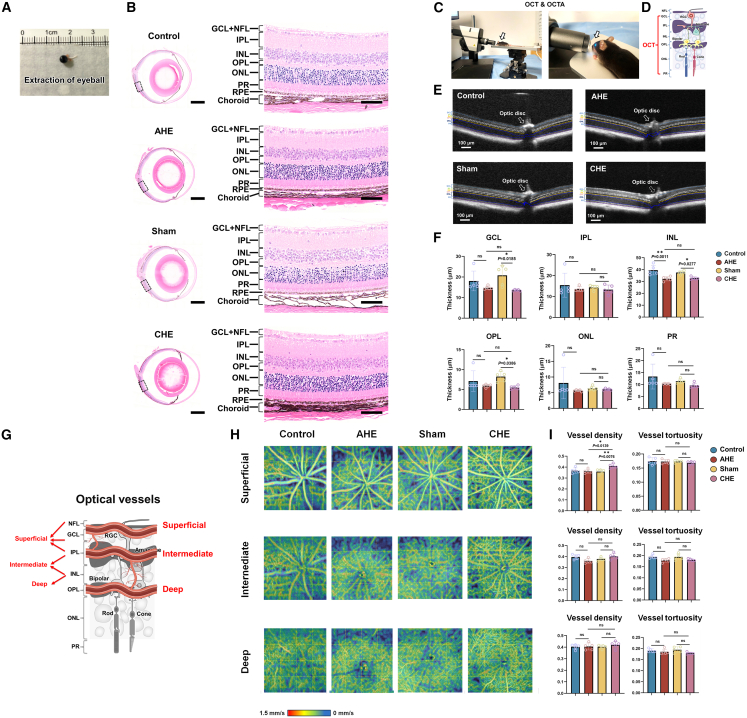
Results of H&E staining, OCT, and OCTA imaging in mouse model of HE (A) Eyeballs were collected from AHE mice on day 4 after TAA injection and from CHE mice on day 15 after BDL surgery. The eyeballs were dissected, the retinas were isolated and fixed, and representative images of the mouse eyes were taken. (B) H&E staining of the whole eyeball (left) and magnified retina (right) in control, AHE, sham, and CHE groups. Scale bars, 500 μm (left) and 100 μm (right). (C) OCT and OCTA examinations were performed on AHE mice at day 4 after TAA injection and on CHE mice at day 15 after BDL surgery, with representative images showing the testing environment. White arrows indicate the mouse placement position. (D) Schematic diagram of retinal structure as detected by OCT. (E) Retinal OCT images of control and AHE groups (scale bar, 100 μm). Retinal OCT images of sham and CHE groups (scale bar, 100 μm). (F) Statistical results of retinal layer thickness detected by OCT. (G) Schematic diagram illustrating the principles of OCTA detection. (H) Representative OCTA images of the superficial, intermediate, and deep vascular networks of the retina in control, AHE, sham, and CHE groups. (I) Statistical results for vessel density and vessel tortuosity in the superficial vascular network (*n* = 5 per group). All data are presented as mean ± standard deviation. ∗*p* < 0.05; ∗∗*p* < 0.01; ns, not significant; IPL, inner plexiform layer; NFL, nerve fiber layer; ONL, outer nuclear layer; PR, photoreceptor layer; RPE, retinal pigment epithelium.

To investigate this, we performed retinal H&E staining and *in vivo* optical coherence tomography (OCT) to assess retinal structural integrity.^33^^,^^34^ Figure 3A showed mouse eyeball isolation, and Figure 3B presents H&E staining of the retina in the normal control, AHE, sham, and CHE groups, with no significant structural changes in AHE, sham, and CHE groups. OCT imaging (Figures 3C–3F) revealed a 15.34% reduction (*p* < 0.01) in inner nuclear layer (INL) thickness in AHE mice, but no changes in the other layers, suggesting retinal abnormalities are not the cause of visual dysfunction in AHE mice. In contrast, CHE mice exhibited significant reductions in ganglion cell layer (GCL), INL, and outer plexiform layer (OPL) thickness, indicating retinal structural changes may contribute to visual dysfunction. Although H&E staining in the CHE group revealed that the GCL, INL, and OPL layers maintained clear structures with orderly cellular organization, the abnormal OCT findings prevent us from completely excluding the possibility that visual impairment in CHE may originate from retinal structural abnormalities. Therefore, we cautiously employed AHE mice for subsequent screening of intracerebral structures and neural regulation.

To explore retinal blood supply abnormalities, we used OCT angiography (OCTA) to evaluate vessel density and tortuosity in the central retinal arteries and veins.^34^ The OCTA images (Figures 3G–3I) showed no significant difference in vessel density in the superficial layer between AHE and normal groups, but the CHE group exhibited a 9.93% increase compared to sham (*p* < 0.01). No significant differences were found in vessel tortuosity or in the intermediate and deep layers between the AHE and normal groups, or the CHE and sham groups.

### Screening strategy and phase 1 screening identified 43 SNr-downstream brain regions as candidate areas potentially involved in the dysfunction of the liver-brain-eye axis

**Figure 4:**
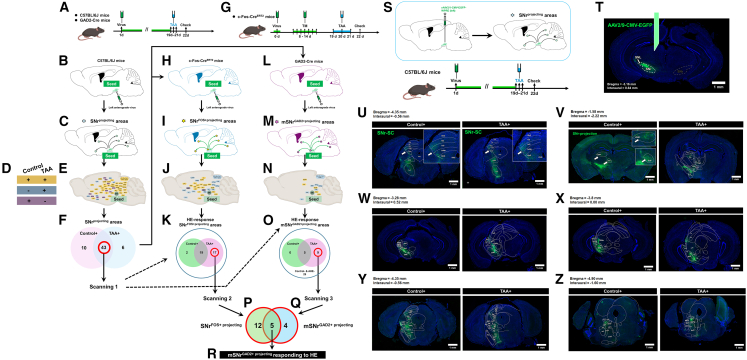
Screening strategy for identifying brain nuclei associated with HE-related visual impairment using SNr as a seed region (A) Schematic workflow of SNr viral injection in C57BL/6J and GAD2-Cre mice. Virus injection was performed on day 1, TAA was injected daily on days 19–21, and samples were collected on day 22. (B) Diagram of left SNr viral injection in C57BL/6J mice. (C) Diagram showing projections from the SNr to downstream regions in C57BL/6J mice (∗ indicates downstream projection areas). (D) “+” indicates the presence of SNr-to-region projections; “−” indicates the absence of such projections. (E) Schematic of specific downstream nuclei receiving projections from the SNr in C57BL/6J mice. (F) Among downstream nuclei, 10 were positive only in the control group, 6 were positive only in the AHE group, and 43 were positive in both groups. (G) Workflow of SNr viral injection in c-Fos-Cre^ERT2^ mice. Virus injection was performed on day 0, tamoxifen was injected daily on days 8–14, TAA was injected daily on days 19–21, and samples were collected on day 22. (H) Diagram of left SNr viral injection in c-Fos-Cre^ERT2^ mice. (I) Diagram showing projections from the SNr to downstream activated regions in c-Fos-Cre^ERT2^ mice (∗ indicates downstream activated projection areas). (J) Schematic of specific downstream regions receiving projections from the SNr in c-Fos-Cre^ERT2^ mice. (K) Among downstream nuclei, 2 were positive only in the control group, 17 were positive only in the AHE group, and 19 were positive in both groups. (L) Diagram of left SNr viral injection in GAD2-Cre mice. (M) Diagram showing downstream nuclei receiving projections from mSNr in GAD2-Cre mice (∗ indicates downstream projection nuclei). (N) Schematic of specific downstream regions receiving projections from mSNr in GAD2-Cre mice. (O) Among activated downstream nuclei, none were positive only in the control group, 9 were positive only in the AHE group, and 5 were positive in both groups. (P) The 17 nuclei activated only in the AHE condition were selected for further analysis. (Q) Intersection of the 9 nuclei activated only in the AHE condition in GAD2-Cre mice and the 17 nuclei activated only in the AHE condition in TRAP mice resulted in the selection of 5 nuclei. (R) These 5 nuclei represent the mSNr^GAD2+^ regions activated under HE conditions. (S) Schematic diagram of SNr viral injection and AHE model construction in C57BL/6J mice. Viral injection was performed on day 1, followed by TAA injections on days 19–21 for 3 consecutive days, and tissue collection on day 22. (T) Diagram of SNr viral injection site (scale bar, 1 mm). (U) Projection maps of SNr downstream nuclei at bregma = −4.35 mm and interaural = −0.56 mm in control and AHE groups (scale bars, 1 mm). (V) Projection maps of SNr downstream nuclei at bregma = −1.58 mm and interaural = −2.22 mm in control and AHE groups (scale bars, 1 mm). (W) Projection maps of SNr downstream nuclei at bregma = −3.28 mm and interaural = 0.52 mm in control and AHE groups (scale bars, 1 mm). (X) Projection maps of SNr downstream nuclei at bregma = −3.8 mm and interaural = 0.00 mm in control and AHE groups (scale bars, 1 mm). (Y) Projection maps of SNr downstream nuclei at bregma = −4.16 mm and interaural = −0.36 mm in control and AHE groups (scale bars, 1 mm). (Z) Projection maps of SNr downstream nuclei at bregma = −4.90 mm and interaural = −1.60 mm in control and AHE groups (scale bars, 1 mm).

In this study, we used the SNr as the seed region for tracing projections to brain nuclei under normal and AHE conditions (Figure 4) according to previous studies.^28^^,^^29^^,^^30^^,^^31^^,^^32^ In phase 1, anterograde labeling virus was injected into the left SNr of C57BL/6J mice, with no injected mice as controls (Figures 4A and 4B). We examined the projection patterns of the SNr to various brain regions (Figure 4C) and categorized the projection areas into three groups (Figure 4D): normal+ and TAA+ projections, normal− and TAA+ projections, and normal+ and TAA− projections. SNr projections common to both normal and TAA groups were identified as Screening Result 1 (Figures 4E and 4F), indicating regions involved in the “liver-brain-eye” axis. In phase 2, c-Fos-Cre^ERT2^ (targeted recombination in active populations [TRAP] mice) were used for anterograde labeling of the SNr (seed region) (Figures 4G and 4H). TRAP mice were used to label SNr projections activated by HE (SNr^FOS+ projecting^) (Figure 4I), leading to Screening Result 2 (Figures 4J and 4K). In phase 3, GAD2-Cre mice labeled medial SNr ^GAD2−positive neurons^ activated by HE (mSNr^GAD2+ projecting^) (Figures 4L and 4M), defining Screening Result 3 (Figures 4N and 4O). The intersection of Screening Results 2 and 3 identified candidate brain nuclei responsible for HE-related pathology (Figures 4P–4R).

In the initial experiment, an anterograde tracer (rAAV2/1-CMV-EGFP-WPRE) was injected into the left SNr of C57BL/6J mice (Figure 4S). Figure 4T confirms the correct viral injection site, and Table 1 summarizes that 53 nuclei received projections from the SNr in the normal group and 49 in the AHE group, with 43 nuclei consistently receiving projections under both conditions; among them, 42 received unilateral projections, 1 received bilateral projections, and 6 received projections only under AHE conditions, while 10 received projections only under normal conditions. Figures 4U–4Z show the rostrocaudal distribution of these double-positive nuclei.

**Table 1:** Using the SNr nucleus as a “seed region,” screening for brain nuclei involved in visual impairments associated with HE was sequentially conducted in wild-type C57BL/6J mice, c-Fos-CreERT2 mice (abbreviated as TRAP mice), and GAD2-Cre mice

	Brain Region	C57BL/6J Control	C57BL/6J AHE	TRAP control	TRAP-AHE	GAD2-Cre-Control	GAD2-Cre-AHE
1	AcbC	_+_	_+_	–	_+_	–	–
2	AcbSh	–	_+_	–	–	–	–
3	APT	_+_	_+_	–	–	–	–
4	APTD	_+_	–	–	–	–	–
5	APTV	_+_	–	–	–	–	–
6	BIC	_+_	_+_	_+_	–	–	–
7	CL	_+_	_+_	_+_	_+_	–	–
8	CM	_+_	_+_	_+_	_+_	–	–
9	CPu	_+_	_+_	_+_	_+_	–	–
10	DMTg	_+_	_+_	–	_+_	–	–
11	DpG	_+_	_+_	–	_+_	–	–
12	DpMe	_+_	_+_	_+_	_+_	_+_	_+_
13	F	_+_	–	–	–	–	–
14	InG	_+_	_+_	–	_+_	–	_+_
15	InWh	_+_	_+_	–	_+_	–	_+_
16	IPACL	_+_	–	–	–	–	–
17	IPACM	_+_	–	–	–	–	–
18	LDDM	_+_	_+_	–	_+_	–	–
19	LDTgV	_+_	_+_	–	_+_	–	–
20	LDVL	–	_+_	–	–	–	–
21	LGP	_+_	_+_	_+_	–	–	–
22	LH	_+_	_+_	_+_	_+_	–	_+_
23	LPB	_+_	_+_	–	_+_	–	–
24	LPMR	_+_	_+_	–	_+_	–	–
25	LSI	_+_	_+_	–	_+_	–	–
26	MCLH	_+_	–	–	–	–	–
27	MD	_+_	_+_	_+_	_+_	–	–
28	MGV	_+_	–	–	–	–	–
29	Mo5	_+_	_+_	–	–	–	–
30	MPB	_+_	_+_	–	–	–	–
31	MS	–	_+_	–	–	–	–
32	MT	_+_	_+_	–	_+_	–	–
33	MZMG	_+_	_+_	_+_	_+_	–	–
34	Op	_+_	_+_	–	_+_	–	_+_
35	PAG	–	_+_	–	–	–	–
36	PIL	_+_	_+_	_+_	_+_	–	–
37	PnO	_+_	_+_	_+_	_+_	_+_	_+_
38	Po	_+_	_+_	_+_	_+_	–	–
39	PoT	_+_	_+_	_+_	_+_	–	–
40	PP	_+_	_+_	_+_	_+_	_+_	_+_
41	PPTg	_+_	_+_	–	_+_	–	_+_
42	PR	_+_	_+_	_+_	_+_	–	–
43	RRF	_+_	_+_	_+_	_+_	–	_+_
44	SG	–	_+_	–	–	–	–
45	SI	_+_	–	–	–	–	–
46	SNc	_+_	_+_	_+_	_+_	_+_	_+_
47	SNL	_+_	_+_	–	_+_	–	_+_
48	SPFPC	_+_	–	–	–	–	–
49	STh	_+_	_+_	_+_	_+_	–	–
50	Su5	_+_	_+_	–	–	–	–
51	Sub	_+_	_+_	–	_+_	–	–
52	SubB	_+_	_+_	_+_	_+_	_+_	_+_
53	SubCD	_+_	_+_	–	–	–	–
54	SubG	_+_	–	–	–	–	–
55	VDB	–	_+_	–	–	–	–
56	VM	_+_	_+_	_+_	_+_	–	–
57	VP	_+_	_+_	–	_+_	–	–
58	VTA	_+_	_+_	–	_+_	–	_+_
59	ZI	_+_	_+_	_+_	_+_	–	_+_

The study utilized both control mice and TAA-induced AHE mice. A “+” symbol indicated that the nucleus receives projections from the seed region, whereas a “−” symbol indicated the absence of such projections.

### Phase 2 further screened 23 SNr-downstream brain regions as potential candidates implicated in the dysfunction of the liver-brain-eye axis

**Figure 5:**
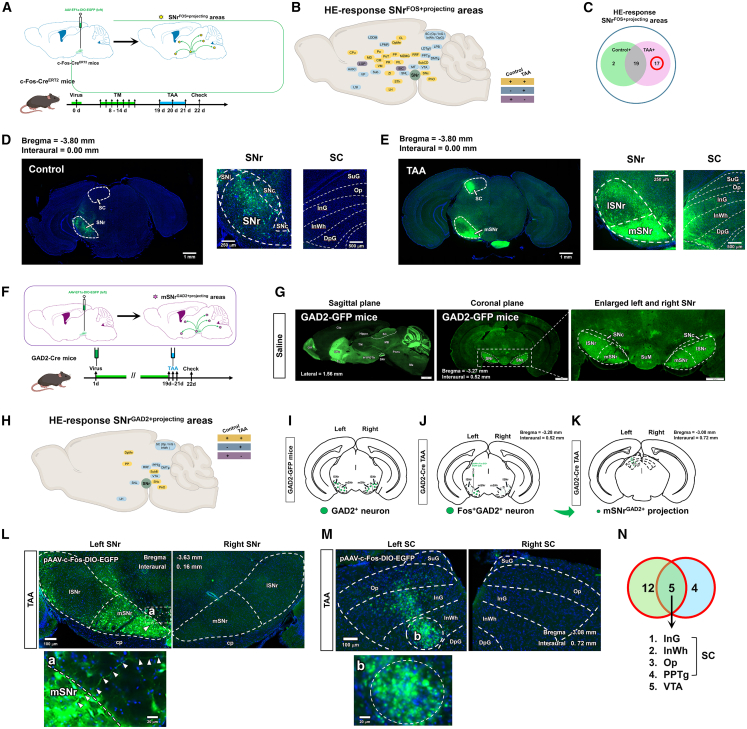
Mapping brain nuclei and investigating mSNrGAD2-SC pathways in HE-related visual impairments using anterograde tracing in TRAP and GAD2-Cre mice (A) Experimental workflow of SNr viral injection and AHE model construction in TRAP mice. Viral injection was performed on day 0, followed by tamoxifen injections on days 8–14 for 7 consecutive days to label SNr-activated neurons. DIO virus was used for tracing downstream pathways. TAA injections were administered on days 19–21 for 3 consecutive days, and tissues were collected on day 22. (B) Specific downstream regions of SNr projections: Yellow, nuclei positive in both control and AHE groups; blue, nuclei positive only in the AHE group; purple, nuclei positive only in the control group. (C) Summary of SNr downstream activated projection nuclei in TRAP mice: 2 nuclei positive only in the control group, 17 nuclei positive only in the AHE group, 19 nuclei positive in both control and AHE groups. (D) Confocal fluorescence images showing SNr viral injection sites and SC projections in control mice; scale bars, 1 mm (left) and 500 μm (right). (E) Confocal fluorescence images showing SNr viral injection sites and SC projections in TRAP mice; scale bars, 1 mm (left) and 500 μm (right). (F) Workflow of SNr viral injection and AHE model construction in GAD2-Cre mice. Viral injection was performed on day 1, followed by TAA injections on days 19–21 for 3 consecutive days and tissue collection on day 22. (G) Confocal fluorescence images in sagittal and coronal sections of GAD2-GFP mice (scale bars: 1 mm and 500 μm). Green fluorescence indicates GABAergic neurons. (H) Specific downstream regions of activated GAD2 projections in GAD2-Cre mice: Yellow, nuclei positive in both control and AHE groups; blue, nuclei positive only in the AHE group; purple, nuclei positive only in the control group. (I) Schematic diagram of GAD2 neuron distribution in the SNr of GAD2-GFP mice. (J) Schematic diagram of activated GAD2 neuron distribution in mSNr of GAD2-Cre mice. (K) Schematic diagram of activated GAD2 neuron distribution in projections from mSNr to SC. (L) Fluorescence images of activated GABAergic neurons in the SNr of GAD2-Cre mice (arrowheads indicate nerve fibers) (scale bars, 100 and 20 μm). (M) Fluorescence images of activated GABAergic neurons in the SC of GAD2-Cre mice (green indicates activated GABAergic neurons) (scale bars, 100 and 20 μm). (N) Intersection of 9 nuclei activated only in AHE in GAD2-Cre mice and 17 nuclei activated only in AHE in TRAP mice identified the following nuclei: InG, InWh, Op, PPTg, and VTA.

To identify neural pathways activated under HE conditions, we combined the TRAP technique with unilateral anterograde tracing, as described previously.^31^ On day 1, a double-floxed inverted open reading frame (DIO)-carrying anterograde tracer virus (rAAV2/1-EF1a-DIO-EGFP) was injected into the SNr of TRAP mice (Figure 5A). From days 8–14, mice received daily tamoxifen injections (75 mg/kg), followed by AHE model induction on days 19–21. On day 22, brain sections were prepared to identify activated nuclei under AHE conditions (Figure 5B). Figure 5C shows the SNr-specific anterograde labeling process, identifying SNr^FOS+ projecting^ regions in response to HE. Figures 5D and 5E show that in the control group, most activated neurons were in the lateral SNr, whereas the AHE group showed activation in both the medial and lateral SNr, with 70% of neurons activated in the medial SNr. This suggests that medial SNr neurons are “seed neurons” for HE-related pathways.

### Phase 3 identified mSNrGAD2-SC pathway as a critical role in the dysfunction of the liver-brain-eye axis

To further investigate, rAAV2/1-*c*-Fos-DIO-EGFP-WPRE virus was used in GAD2-Cre mice to label HE-activated GABAergic neurons (Figure 5F). Results confirmed the presence of GABAergic neurons in the SNr expressing GAD2 (Figure 5G). Anterograde tracing identified SNr projection nuclei positive in the AHE group but negative in controls (Figure 5H). Figures 5I–5K summarize the experimental workflow. Figures 5L and 5M show that both control and AHE groups predominantly activated the medial SNr in GAD2-Cre mice, identifying mSNr^GAD2^ neurons as “seed neurons” projecting to the SC. Tables 1 and 2 detail the projection patterns of mSNr^GAD2^ neurons, identifying five overlapping regions between mSNr^GAD2^ and TRAP mice that are key candidates for mediating HE-related visual abnormalities: intermediate gray layer of the SC (InG), intermediate white layer of the SC (InWh), optic nerve layer of the SC (Op), posterior pretectal nucleus (PPTg), and ventral tegmental area (VTA) (Figure 5N). These regions highlight the potential role of inhibitory mSNr^GAD2^-SC projections in the “liver-brain-eye” network, suggesting a need for further exploration.

**Table 2:** Summary of the screening results for brain nuclei associated with visual impairments in HE, using the SNr nucleus as the “seed region,” sequentially conducted in wild-type C57BL/6J mice, c-Fos-CreERT2 mice (TRAP mice), and GAD2-Cre mice

	C57BL/6J	TRAP	GAD2-Cre
Control+TAA	43	20	5
Unique to the control group	10	2	0
Unique to the TAA group	6	23	9
TAA	49	43	14
Control	53	22	5
Sum	59	45	14

### Chemogenetic inhibition, rather than activation, of mSNrGAD2-SC pathway alleviated visual dysfunction in the AHE model

**Figure 6:**
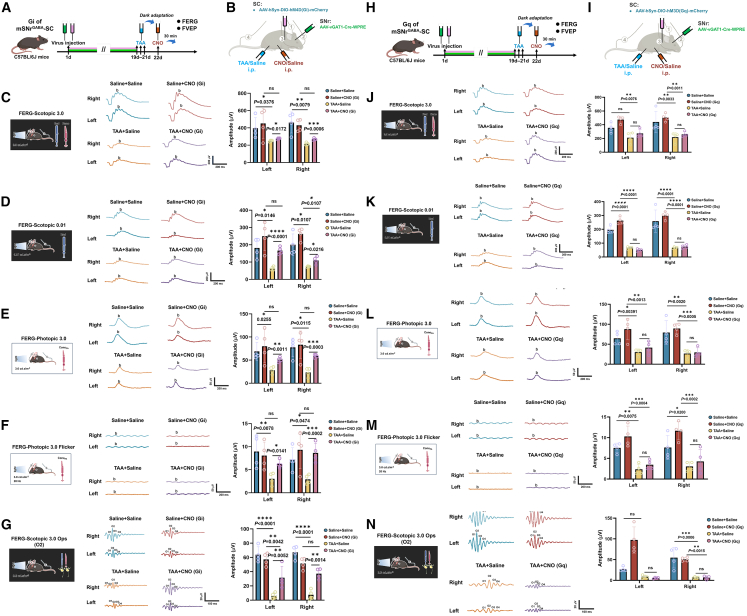
Chemical modulation of SNr-SC GABAergic neurons and ERG results in AHE model mice (A) Viral injection was performed on day 1, followed by TAA injections on days 19–21 for 3 consecutive days. On day 22, CNO was administered, and Flash Visual Evoked Potential (FVEP) and FERG were recorded 30 min later. (B) Schematic representation of the chemogenetic inhibition experiment. (C) Representative FERG-Scotopic 3.0 waveforms in Saline + Saline and Saline + CNO(Gi) groups and representative FERG-Scotopic 3.0 waveforms in TAA + Saline and TAA + CNO(Gi) groups (*n* = 4 per group). (D) Representative FERG-Scotopic 0.01 waveforms in Saline + Saline and Saline + CNO(Gi) groups and representative FERG-Scotopic 0.01 waveforms in TAA + Saline and TAA + CNO(Gi) groups (*n* = 4 per group). (E) Representative FERG-Photopic 3.0 waveforms in Saline + Saline and Saline + CNO(Gi) groups and Representative FERG-Photopic 3.0 waveforms in TAA + Saline and TAA + CNO(Gi) group (*n* = 4 per group). (F) Representative FERG-Photopic 3.0 flicker waveforms in Saline + Saline and Saline + CNO(Gi) groups and representative FERG-Photopic 3.0 flicker waveforms in TAA+ Saline and TAA+ CNO(Gi) groups (*n* = 4 per group). (G) Representative FERG-Scotopic 3.0 Ops waveforms in Saline + Saline and Saline + CNO(Gi) groups and representative FERG-Scotopic 3.0 Ops waveforms in TAA + Saline and TAA + CNO(Gi) groups (*n* = 4 per group). (H) Viral injection was performed on day 1, followed by TAA injections on days 19–21 for 3 consecutive days. On day 22, CNO was administered, and FVEP and FERG were recorded 30 min later. (I) Schematic representation of the chemogenetic activation experiment. (J) Representative FERG-Scotopic 3.0 waveforms in Saline + Saline and Saline + CNO(Gq) groups and Representative FERG-Scotopic 3.0 waveforms in TAA + Saline and TAA + CNO(Gq) groups (*n* = 4 per group). (K) Representative FERG-Scotopic 0.01 waveforms in Saline + Saline and Saline + CNO(Gq) groups and representative FERG-Scotopic 0.01 waveforms in TAA + Saline and TAA + CNO(Gq) groups (*n* = 4 per group). (L) Representative FERG-Photopic 3.0 waveforms in Saline + Saline and Saline + CNO(Gq) groups and representative FERG-Photopic 3.0 waveforms in TAA + Saline and TAA + CNO(Gq) groups.(*n* = 4 per group). (M) Representative FERG-Photopic 3.0 flicker waveforms in Saline + Saline and Saline + CNO(Gq) groups and representative FERG-Photopic 3.0 flicker waveforms in TAA + Saline and TAA + CNO(Gq) groups (*n* = 4 per group). (N) Representative FERG-Scotopic 3.0 Ops waveforms in Saline + Saline and Saline + CNO(Gq) groups and representative FERG-Scotopic 3.0 Ops waveforms in TAA + Saline and TAA + CNO(Gq) groups (*n* = 4 per group). All data are presented as mean ± standard deviation. ∗*p* < 0.05, ∗∗*p* < 0.01, ∗∗∗*p* < 0.001, ∗∗∗∗*p* < 0.0001; ns: not significant vs. sham. Two-tailed, unpaired, Student’s *t* test or all. data.

The manipulation of specific cell populations was achieved by the recently developed chemogenetic approaches. DREADDs are genetically modified muscarinic receptors that can be activated by clozapine N-oxide (CNO), a pharmacologically inert metabolite of the atypical antipsychotic drug clozapine. DREADDs could couple via the Gq or Gi pathways to stimulate or inhibit neuronal activity, respectively. CNO was used to activate the hM4D(Gi)receptor and hM3D(Gq)receptor,^32^ with experimental procedures shown in Figures 6A and 6B.

FERG-Scotopic 3.0 results (Figure 6C) showed that TAA-induced AHE reduced b-wave amplitudes by 37% (*p* < 0.05) and 53% (*p* < 0.01) in the left and right eyes. Chemogenetic inhibition (Gi + CNO + TAA) partially restored amplitudes by 11% (*p* < 0.05) and 21% (*p* < 0.001). FERG-Scotopic 0.01 (Figure 6D) and FERG-Photopic 3.0 (Figure 6E) showed significant reductions in b-wave amplitude and cone function under AHE, with inhibition restoring amplitudes approximately 2-fold. FERG-Photopic 3.0 Flicker results (Figure 6F) revealed 60% reductions in peripheral cone function under AHE, with inhibition restoring amplitudes by over 2-fold. These findings confirm that chemogenetic inhibition of mSNr^GAD2^-SC pathways alleviates photoreceptor dysfunction under AHE conditions. FERG-Scotopic 3.0 oscillatory potentials (Figure 6G) revealed a 90% reduction in signal transmission from photoreceptors to non-photoreceptor cells, with chemogenetic inhibition restoring transmission 6-fold, alleviating retinal signal deficits.

In chemogenetic activation experiments, AHE was induced by TAA, and no significant restoration of b-wave amplitudes or signal transmission was observed with activation (Figures 6H–6N), confirming the ineffectiveness of activating mSNr^GAD2^-SC pathways.

**Figure 7:**
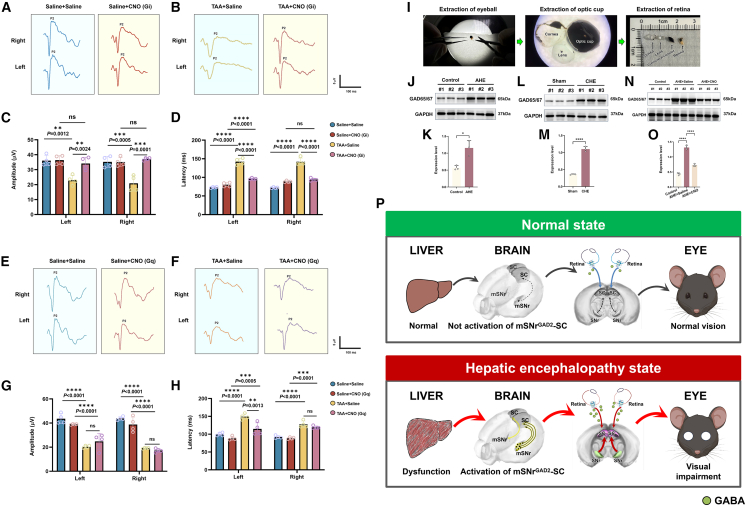
Effects of chemogenetic inhibition/activation of mSNrGAD2-SC projections on VEP and retinal GAD65/67 protein expression in AHE mice (A) Representative VEP waveforms for Saline + Saline and Saline + CNO(Gi) groups (both eyes). (B) Representative VEP waveforms for TAA + Saline and TAA + CNO(Gi) groups (both eyes). (C) Statistical results for VEP P2 amplitude in Saline + Saline, Saline + CNO(Gi), TAA + Saline, and TAA + CNO(Gi) groups: Gi + Saline group (*n* = 4 per group). (D) Statistical results for VEP P2 latency in Saline + Saline, Saline + CNO(Gi), TAA + Saline, and TAA + CNO(Gi) groups: Gi + Saline group (*n* = 4 per group). (E) Representative VEP waveforms for Saline + Saline and Saline + CNO(Gq) groups (both eyes). (F) Representative VEP waveforms for TAA + Saline and TAA + CNO(Gq) groups (both eyes). (G) Statistical results for VEP P2 amplitude in Saline + Saline, Saline + CNO(Gq), TAA + Saline, and TAA + CNO(Gq) groups: Gq + Saline group (*n* = 4 per group). (H) Statistical results for VEP P2 latency in Saline + Saline, Saline + CNO(Gq), TAA + Saline, and TAA + CNO(Gq) groups: Gq + Saline group (*n* = 4 per group). (I) Retinal sampling process in mice, showing sequential steps: enucleation of the eyeball, isolation of the optic cup, and collection of the retina. (J) Western blot analysis showing GAD65/67 protein expression in the retina of control and AHE groups. (K) Quantitative analysis of GAD65/67 protein levels (*n* = 3 per group). (L) Western blot analysis showing GAD65/67 protein expression in the retina of sham and CHE groups. (M) Quantitative analysis of GAD65/67 protein levels (*n* = 3 per group). (N) Western blot analysis showing GAD65/67 protein expression in the retina of control, AHE + Saline, and AHE + CNO groups. (O) Quantitative analysis of GAD65/67 protein levels (*n* = 3 per group). (P) Mechanism diagram of the present scientific hypothesis. All data are presented as mean ± standard deviation. ∗*p* < 0.005, ∗∗*p* < 0.01, ∗∗∗*p* < 0.001; ∗∗∗∗*p* < 0.0001; ns, not significant vs. sham. Two-tailed, unpaired, Student’s *t* test for all data.

Figures 7A and 7B showed typical VEP P2 waveforms for the left and right eyes. Compared to the Gi + Saline group, the Gi + Saline + TAA group showed a 40% reduction in P2 amplitude (*p* < 0.05), confirming visual conduction impairments under AHE. The Gi + CNO + TAA group exhibited a 1.5-fold recovery in P2 amplitude compared to the Gi + Saline + TAA group (*p* < 0.01) (Figure 7C), indicating that chemogenetic inhibition of the SNr-SC projection alleviates abnormalities. No significant differences were found between the Gi + CNO and Gi + Saline groups. Regarding P2 latency, the Gi + Saline + TAA group showed a 2-fold increase in latency (*p* < 0.0001), confirming visual conduction impairments. The Gi + CNO + TAA group showed a one-third reduction in latency (*p* < 0.0001) (Figure 7D), suggesting improvement with chemogenetic inhibition.

Figures 7E and 7F show VEP P2 waveforms for the left and right eyes in the Gq group. The Gq + Saline + TAA group showed a 50% reduction in P2 amplitude (*p* < 0.0001), confirming visual conduction impairments. No significant differences were observed between the Gq + CNO + TAA and Gq + Saline + TAA groups (Figure 7G), indicating no improvement with chemogenetic excitation. No significant differences were found between the Gq + CNO and Gq + Saline groups. Regarding P2 latency, the Gq + Saline + TAA group showed a 1.3-fold increase in latency (*p* < 0.0001). No significant differences were observed between the Gq + CNO + TAA and Gq + Saline + TAA groups (Figure 7H), suggesting no effect of chemogenetic excitation.

### Chemogenetic inhibition, rather than activation, of mSNrGAD2-SC pathway alleviated the elevated GAD65/67 levels in the retina in the AHE model

Western blotting detected retinal γ-aminobutyric acid (GABA) levels, with Figure 7I illustrating the retinal isolation process. Figures 7J and 7K demonstrated that the GABA concentrations in the AHE group were approximately twice those of the control group (*p* < 0.05), suggesting a potential impairment in neurotransmission. Figures 7L and 7M show a 2.5-fold increase in GAD65/67 levels in the CHE group (*p* < 0.0001). Figures 7N and 7O show that GABA levels in the Gi + Saline + TAA group increased by 2-fold (*p* < 0.0001) and decreased by 40% in the Gi + CNO + TAA group (*p* < 0.0001), nearly returning to normal, confirming that chemogenetic inhibition alleviates abnormalities.

## Discussion

Patients with HE often develop visual impairments, representing a “liver-brain-eye” combined injury; however, its underlying mechanism remains elusive. This study validates the hypothesis that liver injury-induced hyperammonemia specifically activates the mSNr^GAD2^-SC pathway, disrupting SC’s regulatory role in visual transmission homeostasis and eventually causing visual impairments (Figure 7P). Under normal liver function (upper panel), ammonia is effectively eliminated, preventing hyperammonemia. The mSNr^GAD2^-SC pathway remains silent, allowing the functional SC to regulate low retinal GABA release. As a result, rods and cones operate normally, and visual signals are accurately transmitted from the retina to the visual cortex, yielding normal vision. In HE (lower panel), liver dysfunction prevents effective ammonia clearance, leading to hyperammonemia. This selectively activates the mSNr^GAD2^-SC pathway. The dysregulated SC loses control over retinal GABA release, leading to excessive GABA production and release into the visual transmission pathway, disrupting visual transmission homeostasis and ultimately resulting in visual impairments.

Our findings delineate a multistep pathway linking hyperammonemia to visual impairment: (1) liver injury leads to hyperammonemia, (2) ammonia specifically activates GAD2-positive GABAergic neurons in the medial SNr, (3) these neurons project to and release GABA into the SC, (4) the aberrant SC activity disrupts its normal modulation of retinal GABAergic tone, and (5) consequently, retinal GABA synthesis and release are elevated, impairing visual signal transmission and resulting in visual deficits.

The pathogenesis of HE is not fully understood, with the ammonia hypothesis still prevailing as the primary explanation for HE mechanisms.^20^^,^^21^^,^^22^ In this study, we utilized acute and chronic HE models as recommended by the International Society of Hepatic Encephalopathy and Nitrogen Metabolism.^38^ Previous research has demonstrated that hyperammonemia is observed in various AHE and CHE animal models.^39^^,^^40^^,^^41^^,^^42^^,^^43^^,^^44^^,^^45^^,^^46^^,^^47^ Our results further confirmed the presence of hyperammonemia in HE model mice.^28^^,^^29^^,^^30^^,^^31^^,^^32^

Extensive prior research has demonstrated that liver disease leads to ocular dysfunction.^5^^,^^6^^,^^7^^,^^8^^,^^9^^,^^10^^,^^11^^,^^12^^,^^13^^,^^14^^,^^15^^,^^16^^,^^17^^,^^18^^,^^19^ In 1981, Varnek L et al.^7^ have documented color vision deficiencies in all 17 patients with HE. In 2010, Krismer F et al.^13^ have examined 70 cirrhotic patients and 31 post-liver transplant patients and established saccade latency as an objective quantitative parameter for HE. In 2005, Montagnese S et al.^9^ have assessed the smooth pursuit eye movement performance of 56 HE patients. In 2015, Cunniffe N et al.^10^ have found significantly prolonged saccade latency in CHE patients compared to non-CHE patients. In 2023, Córdoba CA et al.^18^ have introduced an automated gaze-tracking system capable of accurately diagnosing minimal HE through machine learning analysis of 150 eye movement-related metrics. In 2022, Casanova-Ferrer F et al.^48^ have uncovered multiple ocular dysfunctions in HE patients.

VEP and ERG serve as critical indicators of ocular health by assessing the functionality of retinal photoreceptors and the detailed visual signal transmission from the retina to the visual cortex.^14^^,^^49^^,^^50^^,^^51^ In 1981, Zeneroli M L et al.^49^ have pioneered the use of VEP testing to assess visual transmission in a galactosamine-induced acute liver failure rat model. In 1984, Zeneroli M L et al.^50^ have shown that all HE patients exhibited VEP abnormalities. In 1987, Levy J et al.^52^ have identified significant delays in P2 wave latency among HE patients. In 1990, Davies M et al.^53^ have positioned VEP abnormalities as early HE indicators and long-term monitoring tools. In 1997, Eckstein A et al.^54^ have revealed ERG oscillatory potentials as the most sensitive indicators of HE.

Our research group has previously investigated retinal function and visual signal transmission in various animal models, including rats and mice, using ERG and VEP and has published a series of related studies.^33^^,^^34^^,^^35^^,^^36^^,^^37^ Building upon this foundation, the present study is the first to perform specifically ERG and VEP—in a mouse model of HE. The results demonstrated that under HE conditions, both cone cells in the central retina (extending approximately 1 mm from the ora serrata toward the posterior pole) and peripheral retina (extending from the region adjacent to the central area to the ora serrata), as well as all rod cells, exhibited functional impairment. In addition, we observed transmission deficits from photoreceptors to secondary retinal neurons, as well as conduction abnormalities from the retina to the visual cortex. These findings provide the first direct evidence of visual dysfunction in a murine model of HE. Furthermore, we found that visual perception and signal transmission abnormalities were more pronounced in CHE compared with AHE, which is consistent with clinical observations. The differences in visual dysfunction between the AHE and CHE models reflect the distinct pathophysiological processes of AHE and CHE. In the acute AHE model, the neurotoxic effects of hyperammonemia on the retina and visual pathways are likely transient, leading to milder visual impairments.^13^ Due to the shorter duration of ammonia exposure, retinal damage and neural transmission disruption are less severe, and some degree of recovery may be possible. This aligns with the relatively moderate reductions in FERG and VEP measures observed in AHE animals. In contrast, the chronic CHE model, with prolonged exposure to hyperammonemia, likely leads to more severe and irreversible damage.^55^ Long-term ammonia exposure places continuous stress on retinal cells and visual pathways, exacerbating cellular degeneration, particularly in retinal ganglion cells (RGCs) and photoreceptors. This chronic damage may lead to significant structural changes in the retina, such as thinning of the GCL and INL, both of which are crucial for visual processing. These structural alterations help explain the more severe reductions in FERG amplitudes, as well as the substantial decrease in P2 wave amplitude and increased latency observed in the CHE group. Furthermore, chronic liver dysfunction is often accompanied by heightened inflammatory responses and glial cell activation.^56^^,^^57^ both of which are known to exacerbate retinal dysfunction and neural transmission abnormalities. The persistent neuroinflammatory environment may further damage the integrity of the retina and visual pathways, resulting in more pronounced electrophysiological changes in the CHE animals. In the AHE model, however, the acute inflammatory response may be less pronounced, allowing for partial restoration of retinal function. In summary, the more severe visual dysfunction observed in the CHE model is likely due to the cumulative effects of prolonged hyperammonemia exposure on retinal structure and function. Chronic ammonia exposure may lead to irreversible retinal degeneration and persistent disruptions in visual signal transmission, while the shorter ammonia exposure in AHE allows for less-severe damage and a greater potential for recovery. These findings underscore the importance of the duration and severity of liver dysfunction in determining the extent of visual dysfunction.

In addition, we applied *in vivo* OCTA to visualize the morphological architecture of the superficial, intermediate, and deep retinal capillary plexuses originating from the central retinal artery. To the best of our knowledge, this is the first report to characterize the fine structural features of the retina and its vasculature in an HE mouse model. The results showed that, compared with normal controls, both AHE and CHE groups displayed well-organized retinal layers with clear boundaries, orderly cell alignment, and no evidence of abnormal proliferation or degenerative changes. Likewise, no significant pathological alterations were observed in the retinal capillaries. Histological analysis and OCT/OCTA are not entirely consistent but complementary. Histological analysis provides mechanistic insights into early damage by revealing microscopic pathological changes, such as apoptosis and focal vascular abnormalities. In contrast, OCT/OCTA enables non-invasive, dynamic measurements of structural and blood flow changes *in vivo*, making it more suitable for monitoring overall trends. These differences arise from disparities in detection levels, inherent biases, and temporal dynamics of the two techniques—for instance, acute HE exhibits a “function-structure dissociation,” while chronic HE shows diffuse damage without significant changes in layer thickness. Combining the results of both methods helps to comprehensively understand the pathological processes and evolution of visual function impairment in AHE and CHE models. Collectively, these results suggest that the visual impairment observed in HE is not primarily caused by retinal pathology itself, but rather may originate from abnormalities within central visual processing nuclei in the brain.

The SN includes the pars compacta (SNc) and SNr. SNc mainly comprises dopaminergic neurons, famously implicated in Parkinson’s disease due to neuronal loss, while SNr is primarily composed of GABAergic neurons.^23^^,^^24^^,^^25^^,^^26^^,^^27^ Studies by Cauli O et al.^58^ and Gálvez-Gastélum FJ et al.^59^ have revealed that GABAergic neurotransmission imbalance within the SNr is involved in HE in animal models. Later studies^60^^,^^61^^,^^62^^,^^63^^,^^64^ have systematically reviewed the molecular mechanisms underlying this imbalance and its role in HE. In 2014, Butterworth RF et al.^65^ have reviewed structural brain lesions in HE patients, highlighting significant associations with the thalamus, cerebellum, globus pallidus, and SN. Given that abnormalities in these structures are common in tremor-associated diseases, they proposed the term *cirrhotic Parkinson’s syndrome*. Extensive clinical research^65^^,^^66^^,^^67^^,^^68^ has demonstrated that HE patients, particularly those with surgically or spontaneously induced portosystemic shunts, frequently show T1 hyperintensity in the SN on MRI. In 2024, Wang M et al.^69^ have applied quantitative susceptibility mapping to assess brain iron deposition in 25 HE patients, identifying a significant correlation between SN iron deposits and higher-order cognitive impairments.

In our previous studies,^28^^,^^29^^,^^30^^,^^31^^,^^32^ we found that the SNr specifically responds to hyperammonemic conditions in the brain, as evidenced by three major findings. First, under TAA-induced AHE, FOS immunostaining demonstrated selective activation of GABAergic neurons within the SNr, predominantly those expressing GAD2 in its medial subregion. Second, local administration of ammonium chloride into the SNr, which mimics region-specific hyperammonemia, led to increased FOS expression in SNr GABAergic neurons. Third, chemogenetic inhibition of GAD2-positive neurons in the medial SNr effectively alleviated motor deficits in HE mice, whereas chemogenetic activation of these neurons in normal mice induced HE-like motor impairments. Based on these findings, the present study used the SNr as a seed region to trace its downstream projections under AHE conditions. Our experimental strategy consisted of three stages, conducted sequentially in wild-type C57BL/6J mice, c-Fos-Cre^ERT2^ mice (hereafter referred to as *TRAP mice*, which, when combined with DIO viral vectors, enable specific labeling of AHE-activated neurons), and GAD2-Cre mice (which, when combined with DIO viral vectors, allow selective labeling of AHE-activated GABAergic neurons). Using the TRAP technique in combination with anterograde tracing,^70^^,^^71^ we precisely labeled neurons activated during HE, in which Cre expression is induced within a tamoxifen injection time window. This approach, together with chemogenetic manipulation,^72^^,^^73^^,^^74^^,^^75^^,^^76^ allowed us to verify the functional role of the *mSNr*^*GABA*^*-SC* pathway in AHE-associated visual dysfunction. Through this strategy, we identified five downstream nuclei—*InG*, *InWh*, *Op*, *PPTg*, and *VTA*—as candidate regions potentially involved in HE-related visual abnormalities. Among them, *Op*, *InG*, and *InWh* correspond to the third, fourth, and fifth layers of the *SC*, respectively, whereas *PPTg* and *VTA* have not been previously reported to participate in visual signal transmission. Therefore, we propose a scientific hypothesis that the inhibitory projection from mSNr^GAD2^-SC pathway may represent a key neural circuit underlying the liver-brain-eye pathological connection in HE.

The SC is a conserved brain structure in mammals, essential for visual regulation.^76^^,^^77^^,^^78^^,^^79^^,^^80^^,^^81^^,^^82^^,^^83^^,^^84^^,^^85^^,^^86^ It integrates visual, auditory, and somatosensory inputs to govern reflexive and cognitive behaviors, such as eye and head movements and escape responses. Advances in genomics and functional studies have identified diverse neuronal subtypes within the SC, including wide-field vertical cells, inhibitory neurons, and Pitx2-positive neurons, each with specific functions. Research demonstrates that the SC receives afferents from peripheral RGCs and the central SNr. Optogenetic studies in animals reveal that SC stimulation markedly mitigates RGC loss in glaucoma models, with neuroprotection primarily mediated via retrograde signaling rather than direct RGC activation.^82^ Prior research highlights that GABAergic projections from the SNr to the SC are involved in multiple ocular-related brain functions.^87^^,^^88^^,^^89^^,^^90^ Furthermore, the SNr maintains strong inhibitory control over the SC, preventing unnecessary saccades; its “disinhibition” triggers rapid saccadic movements.^89^^,^^90^ This study shows that during HE state, impaired liver function leads to ineffective ammonia clearance and hyperammonemia, specifically activating the mSNr^GAD2^-SC pathway. This dysfunctional SC loses its regulatory control over retinal GABA release, leading to excessive GABA synthesis and release into the visual pathway. Consequently, this disrupts transmission homeostasis and ultimately results in visual impairment.

### Limitations of the study

This study has several limitations. The molecular mechanisms underlying the retina-SC projection pathway have not been elucidated. Furthermore, the contribution of excitatory neuronal populations to HE-associated visual impairment has not been investigated. In addition, the exclusion of CHE model caused by the BDL surgery is still not really clear and limits the understanding of the visual loss pathways.

## Resource availability

### Lead contact

Further information and requests for resources should be directed to and will be fulfilled by the lead contact, Yayun Wang (wangyy@fmmu.edu.cn).

### Materials availability

This study did not generate new unique reagents. All materials used in this study are either commercially available or available from the lead contact upon reasonable request. Any restrictions on material availability are due to institutional or regulatory requirements.

### Data and code availability

• Data: All data reported in this paper will be shared by the lead contact upon request.
• Code: This paper does not report original code.
• Other items: Any additional information required to reanalyze the data reported in this paper is available from the lead contact upon request.

## Acknowledgments

This project was supported by the Medicine Program of 10.13039/501100007547Air Force Military Medical University (JJ24JH09 and 2024JC003) to Y. Wang, the Shaanxi Basic Research Program of Natural Sciences (2024JC-ZDXM-60) to Y.Y., the Xi-jing Hospital Boosting Fund (XJZT24CY15, XJZT2025KX01) to Y.Y., the 10.13039/501100001809National Natural Science Foundation of China (82570679) to Y.Y., and the 10.13039/501100001809National Natural Science Foundation of China (82201627) to F.W.

## Author contributions

Y. Wang and Y.Y. designed the study and interpreted the results. Z.Z. provided the visual-related technical platform. K.L. and Z.W. conducted the animal experiments. Shaoheng Li organized the results. F.W., Y.B., Shujiao Li, C.Z., Z.N., S.Z., Y. Wu, F.T., N.L., T.C., C.R., and others participated in the experiments.

## Declaration of interests

The authors declare no competing interests.

## STAR★Methods

### Key resources table

**Table:** 

REAGENT or RESOURCE	SOURCE	IDENTIFIER
**Antibodies**		

Rabbit anti-GAD65 + 67	Abcam	Cat# ab183999; RRID:AB_3662875
Mouse anti-GAPDH	Abcam	Cat# ab8245; RRID:AB_2107448
HRP-conjugated goat anti-mouse	Abbkine	Cat# A21010; RRID:AB_2728771
HRP-conjugated goat anti-rabbit	Abbkine	Cat# A21020; RRID:AB_2876889

**Bacterial and virus strains**		

rAAV2/1-EF1a-EGFP-WPRE	OBIO	This paper
rAAV2/1-EF1a-DIO-EGFP-WPRE	OBIO	This paper
rAAV2/1-Fos-DIO-EGFP-WPRE	OBIO	This paper
AAV2/9-VGAT1-CRE-WPRE-PA	OBIO	This paper
AAV2/9-hSyn-DIO-hM3D(Gq)-mCherry	OBIO	BC-0182
AAV2/9-hSyn-DIO-hM4D(Gi)-mCherry	OBIO	BC-0236

**Chemicals, peptides, and recombinant proteins**		

Tamoxifen	Sigma	Cat# T5648
Sodium pentobarbital	Sigma	Cat# 1031001
TAA	Sigma	Cat# BCCD3910
Paraformaldehyde phosphate-buffered solution	HaoKebio	Cat# HK2003
ALT	Nanjing Jiancheng	Cat# C009-2-1
AST	Nanjing Jiancheng	Cat# C010-2-1
Ammonia	Nanjing Jiancheng	Cat# A086-1-1
FAS	Servicebio	Cat# G1109-100 ML
Pentobarbital sodium	Sigma-Aldrich	Cat# 1031001
Somnopentyl II	Shengda Animal Pharmaceuticals	Cat# 20230410
Compound tropicamide eye drops	Xingji Pharmaceutical Co	Cat# 230106
Proparacaine hydrochloride eye drops	Santen Pharmaceutical Co	Cat# 563788
levofloxacin eye drops	Santen Pharmaceutical Co	Cat# CV2199

**Experimental models: Organisms/strains**		

c-Fos-CreERT2	Jackson Laboratory	Stock #030323
GFP-Mito	GemPharmatech	N/A
GAD2-ires-cre	Jackson Laboratory	N/A
C57BL/6J	The Fourth Military Medical University	RRID:MGI:3028467

**Software and algorithms**		

Microplate reader	TECAN Spark	https://lifesciences.tecan.cn/multimode-plate-reader
Digital imaging system	Olympus	https://www.olympus.co.uk/
MonPack 3	Metrovision	https://metrovision.fr/
Robotrak	Nanjing Besivision Medical	https://robotrak.cn/zh/homepage
GraphPad Prism 8.02	GraphPad Prism	https://www.graphpad.com/
SPSS 26.0	SPSS	https://spss.softonic.ru/
Fiji	NIH	http://fiji.sc; RRID:SCR_002285
Photoshop	Photoshop	https://www.adobe.com/products/photoshop.html
Fusion FX EDGE	N/A	http://www.vilber.cn/productshow_10.html

### Experimental models and study participant details

#### Animal study

This study utilized male C57BL/6J mice and four transgenic mouse strains (c-Fos-Cre^ERT2^, GAD2-Cre, GFP-Mito, and GAD2-Mito-GFP), aged 6–8 weeks, with body weights of 18–22 g. All experiments followed the ethical guidelines established by the International Association for the Study of Pain and were approved by the Pain Research Committee at the Fourth Military Medical University. Mice were group-housed under standard specific pathogen-free conditions with a 12-h light/dark cycle, controlled temperature (22 ± 1°C) and humidity (50 ± 10%), and had *ad libitum* access to food and water. All mice were randomly allocated to experimental groups.

The C57BL/6J mice were supplied by the Animal Experiment Center of the Fourth Military Medical University(IACUC:20190107).

The c-Fos-Cre^ERT2^ transgenic mice (designated as TRAP) were obtained from Jackson Laboratory (FOS^tm2.1 (icre/ERT2) Luo/J^; stock number #030323), aged 6–8 weeks. This mouse model was designed to express tamoxifen (TM)-inducible enhanced Cre recombinase (icre/ERT2) under the control of the FOS promoter/enhancer elements, without disrupting endogenous Fos expression. TRAP mice are a robust Cre-lox tool. When infected with a virus containing DIO inverted open reading frame, Fos+ neurons undergo tamoxifen-induced iCre recombination, resulting in EGFP expression. These EGFP-positive neurons mark those activated by specific stimuli. The genotyping primers are: GTC CGG TTC CTT CTA TGC AG; GAA CCT TCG AGG GAA GAC G; and CCT TGC AAA AGT ATT ACA TCA CG.

GAD2-ires-cre transgenic mice (referred to as GAD2-Cre) were obtained from Jackson Laboratory, aged 6–8 weeks. In GAD2-Cre mice, the 3′ untranslated region (UTR) of the GAD2 gene is flanked by an endogenous internal ribosome entry site (IRES) and the Cre recombinase gene. Under resting conditions, the GAD2 gene remains inactive. Upon infection with a virus carrying a double-floxed inverted open reading frame (DIO), GAD2 expression is induced. The genotyping primers are: CTTCTTCCGCATGGTCATCT, CACCCCACTGGTTTTGATTT, and AAAGCAATAGCATCACAAATTTCA.

GFP-Mito transgenic mice were produced by GemPharmatech (Nanjing, China), aged 6–8 weeks. The Mito-Tag cassette (CAG-LSL-GFP-Mito tag) was inserted between floxed sequences, and the construct CAG-loxP-STOP-loxP-Kozak-GFP-Mito-TGA-pA was targeted to the Rosa26 locus in the mouse genome. The genotyping primers used were: CCCAAAGTCGCTCTGAGTTGTTA and TGGCGTTATCTATGGGAACATACGTC.

GFP-Mito mice were bred with GAD2-Cre mice to create GAD2-Mito-GFP transgenic mice, aged 6–8 weeks. In these mice, GFP is specifically targeted to the mitochondrial compartments of all GABAergic neurons that express GAD2 in the central nervous system. The genotyping primers used were: CCCAAAGTCGCTCTGAGTTGTTA, TGGCGTTATCTATGGGAACATACGTC, and TCGGGTGAGCATGTCTTTAATCT.

In summary, we employed C57BL/6J, TRAP, GAD2-ires-Cre, and GAD2-Mito-GFP mice. C57BL/6J mice were used to establish TAA and BDL models, as well as to perform visual function assessments. Furthermore, we utilized TRAP mice, in combination with unilateral anterograde viral tracing, to identify specific neural pathways activated under conditions of HE Subsequently, GAD2-Mito-GFP mice were employed to characterize neuronal subtypes within the SNr, demonstrating that this nucleus predominantly consists of inhibitory neurons. Finally, GAD2-ires-Cre mice were used to confirm that GAD2-expressing neurons projecting from the medial SNr to the SC are selectively activated during HE.

The animal species and experimental procedures are detailed in the Table S1.

#### Influence of sex on the study

This study utilized only male mice to circumvent potential confounding effects of the female estrous cycle on neurophysiological and behavioral measurements in pain and hepatic encephalopathy models. Consequently, the findings are explicitly applicable to males, and potential sex-dependent differences in the observed mechanisms remain to be investigated.

#### Establishment of AHE mouse model

This model was designed to induce HE by administering TAA (Sigma, Lot# BCCD3910, USA, 150 mg/kg body weight) intraperitoneally daily for three consecutive days. The control group received an equivalent volume of saline.

#### Establishment of CHE mouse model

Male mice aged 10–12 weeks were anesthetized with sodium pentobarbital (40 mg/kg, intraperitoneal injection) (Sigma, 1031001, USA) for BDL surgery. Prior to surgery, the skin was prepared and disinfected with 70% ethanol, followed by a midline abdominal incision of approximately 1.5 cm. Upon opening the abdominal cavity, the bile duct was separated from the portal vein and hepatic artery. The upper part of the common bile duct was ligated with 5-0 suture to ensure effective occlusion without transection. The abdominal organs were flushed with 0.9% NaCl solution, repositioned, and the abdominal cavity was subsequently sutured and disinfected. In the sham-operated group, the bile duct was isolated without ligation. All mice were observed for up to two weeks. In the BDL model, meloxicam (5 mg/kg) was administered as a prophylactic analgesic prior to surgery and continued to be given once on the third day following the surgical procedure.

### Method details

#### Study design

This study was designed as an experimental animal investigation to elucidate the mechanisms underlying visual dysfunction in HE, with a particular focus on the liver–brain–eye axis and the role of the SNr. Both **AHE** and **CHE** mouse models were established to capture disease-stage–dependent alterations.

Male C57BL/6J mice and multiple transgenic mouse lines, including TRAP, GAD2-ires-Cre, GFP-Mito, and GAD2-Mito-GFP mice, were employed to enable multimodal analyses combining behavioral assessments, visual electrophysiology, retinal imaging, histopathology, viral tracing, and chemogenetic manipulation. All animals were randomly allocated to experimental groups, and all procedures were conducted in accordance with ethical guidelines approved by the Pain Research Committee of the Fourth Military Medical University.

The overall experimental workflow consisted of (1) establishment and validation of HE models, (2) assessment of neurological and emotional phenotypes, (3) evaluation of retinal structure and function *in vivo*, (4) identification of HE-activated neural circuits, and (5) causal manipulation of specific SNr–SC inhibitory pathways.

#### Literature search

No formal literature search or database retrieval was performed as part of the experimental workflow. However, experimental design, selection of animal models, behavioral paradigms, visual electrophysiological protocols, and retinal imaging techniques were informed by established and widely accepted methodologies in the fields of hepatic encephalopathy, visual neuroscience, and systems neurobiology. All experimental procedures followed standardized protocols and international guidelines (e.g., ISCEV standards for visual electrophysiology) to ensure reproducibility and comparability with previous studies.

#### Study selection

Animal selection and experimental grouping were conducted according to predefined criteria. Male mice aged 6–8 weeks (for most experiments) or 10–12 weeks (for BDL surgery) with body weights of 18–22 g were included. Wild-type C57BL/6J mice were used to establish TAA-induced AHE and BDL–induced CHE models and to perform behavioral, electrophysiological, and retinal imaging assessments.

TRAP mice were selected for activity-dependent neuronal labeling combined with anterograde viral tracing to identify neural pathways activated under HE conditions. GAD2-Mito-GFP mice were used to characterize neuronal subtypes within the SNr, whereas GAD2-ires-Cre mice were utilized to selectively label and manipulate GAD2-expressing neurons projecting from the medial SNr to the SC. Sham-operated or saline-treated mice served as controls where appropriate.

#### Data extraction

Data acquisition was performed using standardized and validated experimental protocols. Liver function and HE status were confirmed by measuring serum ALT, AST, and ammonia levels. Neurological impairment and emotional alterations were evaluated using the mNSS, OFT, and EPM.

Visual function was assessed using *in vivo* ERG and **VEP** in strict accordance with ISCEV standards. Retinal structural integrity and vascular architecture were evaluated using **spectral-domain OCT** and **OCTA**. Histological data were obtained from H&E–stained liver and ocular sections. Molecular data were extracted from retinal tissues using western blot analysis.

All electrophysiological, imaging, behavioral, and biochemical data were collected in a blinded manner and recorded in predefined data sheets for subsequent analysis.

#### Data integration

Data from behavioral tests, visual electrophysiology, retinal imaging, histological analyses, viral tracing, and chemogenetic experiments were integrated to construct a comprehensive framework describing HE-induced visual dysfunction. Functional outcomes (ERG and VEP parameters) were correlated with retinal structural changes (OCT and OCTA metrics) and neural circuit activation patterns identified through c-Fos–dependent labeling and anterograde tracing.

Chemogenetic inhibition or activation of GAD2-positive mSNr–SC projections was used to establish causal links between circuit activity and visual impairment. Quantitative analyses were performed using SPSS 26.0 and GraphPad Prism 8.02. Normally distributed data were analyzed using t-tests or one- and two-way ANOVA with appropriate post hoc tests, while categorical data were analyzed using Fisher’s exact test or Pearson’s chi-square test. All data are presented as mean ± standard deviation, and statistical significance was defined as *p* < 0.05.

#### Liver function and ammonia testing

Serum samples were collected to measure ALT and AST levels for assessing liver function damage and to determine ammonia levels for confirming the HE state. ALT (Nanjing Jiancheng, C009-2-1, China), AST (Nanjing Jiancheng, C010-2-1, China), and ammonia (Nanjing Jiancheng, A086-1-1) levels were determined using enzyme-linked immunosorbent assay (ELISA) kits. Data were acquired using a microplate reader (TECAN Spark, Switzerland).

#### Liver and ocular H&E staining

Liver and eye specimens were collected from mice for H&E staining to evaluate structural integrity. Mice were euthanized following anesthesia, and liver and ocular tissues were promptly harvested. Liver tissues were fixed in 4% paraformaldehyde phosphate-buffered solution (HK2003, HaoKebio, China) at 4°C for 48 h, while ocular tissues were fixed in FAS eyeball fixation solution (G1109-100 ML, Servicebio, China) under the same conditions. The fixed tissues were dehydrated, embedded in paraffin, and sectioned into consecutive slices with a thickness of 4 μm. H&E staining was conducted using an H&E staining kit (Vector Labs, USA). After staining, the sections were mounted using neutral resin. Images of the liver and retinal sections were acquired using a digital imaging system (DP71, Olympus, Japan).

#### Modified neurological severity score system

The mNSS evaluates neurological deficits in mice, comprising the tail suspension test (3 points), straight-line walking test (3 points), sensory test (2 points), balance beam test (6 points), and assessment of reflex loss and abnormal movements (4 points). The total score is 18, with higher scores indicating more severe neurological impairment. Specifically, a score of 0 represents normal mice, scores of 1–6 denote mild injury, scores of 7–12 indicate moderate injury, and scores of 13–18 signify severe injury.

#### Open field test

The OFT apparatus consists of an image acquisition system and an operation analysis system(CleverSys Inc, USA), with the experiment conducted in an open-field box (50 cm × 50 cm × 45 cm). The box is surrounded by standard animal dark lighting to maintain a quiet environment. All mice were placed into the open-field box 1 h after the start of the experiment. Mice were positioned in the center of the box, and spontaneous activity was recorded for 10 min, with primary observational indices including total distance traveled (cm), speed (mm/s) and Activities. After each trial, the walls and floor of the open field were cleaned.

#### Elevated plus maze

The EPM apparatus(Ugo Basile, Italy) consists of two open arms (30 cm × 5 cm) and two closed arms (30 cm × 5 cm × 15 cm), elevated 55 cm above the ground. The intersection of the arms forms a neutral zone. A camera is fixed on a stand directly above the EPM. Mice are placed in the neutral zone facing the open arm opposite to the experimenter’s position, and are then allowed to explore freely for 10 min. With observational indices including: total distance traveled (cm), the amount of time spent in the open arms, entry into open arms. In HE mice, the EPM test is used to assess anxiety-like behavior and emotional alterations. The experimental principle is based on the innate avoidance behavior of mice (such as avoiding risks and preferring safe locations). Mice with higher anxiety levels tend to remain in the enclosed arms, whereas spending more time in the open arms indicates a lower degree of anxiety. Its purpose is to reveal the neuropsychiatric dysfunction induced by HE and to evaluate the efficacy of interventions in restoring emotional function.

#### Injection of anterograde labeling virus

After anesthetizing the mice, they were secured on a stereotaxic apparatus. The adeno-associated virus rAAV2/1-EF1a-EGFP-WPRE (OBIO company) was injected into the SNr at coordinates 1.5 mm lateral to the bregma, 3.4 mm posterior, and 4.55 mm deep: X = −1.5 mm; Y = −3.4 mm; Z = −4.55 mm. A total of 400 nL was injected at 80 nL/min into the left side. After injection, the needle remained in place for 10 min before being slowly withdrawn. The scalp was sutured after the surgery. The steps for anterograde labeling virus injection in c-Fos-Cre^ERT2^ mice are generally similar to those for C57BL/6J mice, with two key differences: the virus used is rAAV2/1-EF1a-DIO-EGFP-WPRE (OBIO company), and tamoxifen (75 mg/kg, Sigma, T5648, USA) is administered intraperitoneally every 24 h from days 8–14 post-injection to activate Cre^ERT2^ protein in the brain. Starting on day 19, TAA is injected intraperitoneally for three consecutive days to establish an AHE model. For GAD2-Cre mice, the procedure for anterograde labeling virus injection is also similar to that for C57BL/6J mice, with the primary difference being the use of rAAV2/1-Fos-DIO-EGFP-WPRE (OBIO company).

#### *In vivo* retinal visual electrophysiology examination

Mice were kept in a standard darkroom for 12 h for acclimatization before the experiment. At the beginning of the experiment, systemic anesthesia was induced by intraperitoneal injection of pentobarbital sodium (50 mg/kg, Sigma-Aldrich, 1031001, USA) and 10% Somnopentyl II (0.025 mL/kg, Shengda Animal Pharmaceuticals, 20230410, China). Compound tropicamide eye drops (5 mg/mL, Xingji Pharmaceutical Co., 230106, China) were applied to dilate the pupils of mice, ensuring consistent light entry to avoid variability in results. Proparacaine hydrochloride eye drops (4 mg/mL, Santen Pharmaceutical Co., 563788, Japan) were applied for ocular surface anesthesia. Recording electrodes were positioned on the surface of both corneas. Reference electrodes were inserted 0.5 cm percutaneously into the bilateral cheeks to minimize skin surface electrical signal interference. A ground electrode was placed 0.5 cm into the mouse tail base to prevent interference from ambient AC signals. Visual function testing for all groups was performed using the MonPack 3 visual electrophysiology system (Metrovision, France), strictly adhering to ISCEV standards.

The mouse eyeball first receives white light stimulation at an intensity of 0.01 cd s/m^2^ from a distance of 30 cm, exclusively activating the rod cells in the retina (FERG-scotopic 0.01). Corneal recording electrodes synchronously capture electrical signals. Twenty stimulations are delivered within 1 min to record ERG (b-wave only). A reduction in b-wave amplitude suggests impaired rod cell function in the retina. Next, FERG-scotopic 3.0 is performed by stimulating the mouse retina with white light at an intensity of 3.0 cd s/m^2^, simultaneously activating rod and cone cells. Corneal electrodes record synchronized electrical potentials. Twenty stimulations are delivered within 1 min to capture ERG (a- and b-waves). A reduction in b-wave amplitude indicates dysfunction in both rod and cone cells. FERG-scotopic 3.0 oscillatory potentials are tested by stimulating the mouse retina with flickering white light at an intensity of 3.0 cd s/m^2^ and a frequency of 20 Hz, activating cells beyond rods and cones, such as amacrine cells, horizontal cells, Müller cells, bipolar cells, and ganglion cells. Corneal electrodes record synchronized electrical potentials. Twenty stimulations are applied in 1 min to capture ERG (O1, O2, O3, and O4 waves). Given the variability in O1, O3, and O4 waves, O2 wave amplitude is analyzed, where a reduction suggests impaired photoreceptor-to-non-photoreceptor cell signal transmission. Next, FERG-photopic 3.0 is assessed. The mouse is exposed to white light for 10 min to inhibit rod cell electrical signals, followed by white light stimulation of the retina at an intensity of 3.0 cd s/m^2^ (activating only cone cells). Corneal electrodes capture synchronized electrical signals. Twenty stimulations are delivered within 1 min to record ERG (b-wave only). A decreased b-wave amplitude indicates cone cell dysfunction in the central retina (spanning approximately 1 mm from the ora serrata to the retinal posterior pole). FERG-photopic 3.0 flicker is conducted. The mouse retina receives flickering light stimulation at 3.0 cd s/m^2^ and 10 Hz (activating only cone cells). Corneal electrodes record synchronized electrical signals. Sixty stimulations are applied to capture ERG (a- and b-waves). A reduction in b-wave amplitude indicates cone cell dysfunction in the peripheral retina (from regions adjacent to the central retina to the ora serrata).

#### *In vivo* visual evoked potentials

After ERG testing, corneal recording electrodes are removed, and a recording electrode is inserted at a 45-degree angle into the skin 0.5 cm above the foramen magnum. Reference and ground electrodes are positioned as in ERG testing. The left eye is covered with black light-blocking tape, and light stimulation is applied to the right eye to record its VEP. The process is repeated for the left eye after switching the tape. The mouse eyes receive white light stimulation at an intensity ≥3.0 cd s/m^2^. Fifty stimulations are delivered in 1 min to record VEP graphs, including N2 and P2 waves. P2 wave latency and amplitude are analyzed; prolonged latency and reduced amplitude suggest transmission defects in the neural pathway from the retina to the visual cortex. Upon completion of measurements, all electrodes are removed, and 0.5% levofloxacin eye drops (Santen Pharmaceutical Co., CV2199, Japan) are administered to the mouse’s eyes to prevent infection. The mouse is placed on a heating pad for recovery and then returned to its cage.

#### *In vivo* retinal optical coherence tomography

After the mice were successfully anesthetized, they were positioned on a dedicated OCT mouse platform, and the scanning probe was placed 0.5–1.0 cm from the cornea. Images were sequentially captured from the left and right pupils for 1 min each. OCT acquired a 2.0 × 2.0 mm region centered on the ONH, with a pixel resolution of 600 × 600 × 1280. Using eye-tracking technology, images were captured from both the central and peripheral areas of the animal fundus (Robotrak; Nanjing Besivision Medical Technology Co., China), and approximately 800 images were compiled to produce the OCT imaging. In the OCT images, the nerve fiber layer, inner plexiform layer (IPL), outer plexiform layer, and retinal pigment epithelium were visualized as high-reflective regions, appearing white; the GCL, INL, outer nuclear layer, inner segment layer, outer segment layer, and choroidal layer were identified as low-reflective regions, appearing black. The boundary between the inner and outer segment layers was observed as a high-reflective region, appearing white.

#### *In vivo* retinal capillary network optical coherence tomography angiography

Upon completing OCT recording, the scanning probe was maintained in its position, and the program was set to the OCTA detection mode. A swept-source laser with a 1060 nm central wavelength was utilized, with an A-scan rate of 200 kHz. The scanning pixel data block size was 600 × 600 × 1280 × 20, where each A-scan consisted of 1280 pixels, each B-scan included 600 A-scans, and each C-scan incorporated 600 B-scans. Each B-scan was repeated 20 times. Sequential imaging was conducted for the left and right pupils of the mice, with each session lasting 10 min. Dynamic images of the retinal vascular structures were acquired *in vivo*, encompassing the superficial, middle, and deep capillary networks. Approximately 800 images were fitted to generate the OCTA images. The OCTA images were analyzed to evaluate the vascular skeleton density and tortuosity. Skeleton density = vascular skeleton area/total image area (unit: %). This value indicates the extent of vascular supply. Vascular tortuosity = vascular centerline length/chord length (unitless), representing the complexity and irregularity of vascular morphology. Normally, straighter vessels exhibit tortuosity values near 1, while increased curving raises the value. All data were processed quantitatively using the analysis software provided with the Robotrak small-animal ophthalmic multimodal imaging system. Retinal layers in the SS-OCT images were initially segmented semi-automatically using a custom MATLAB R2017a algorithm and then manually refined by trained researchers.

#### Chemogenetic inhibition and activation

After anesthetizing the mice, they were positioned on a stereotaxic apparatus, and the adeno-associated virus AAV2/9-VGAT1-CRE-WPRE-PA (OBIO company) was injected into the bilateral mSNr. This virus specifically labels GAD2-positive neurons and induces the expression of Cre recombinase. Concurrently, the chemogenetic virus AAV2/9-hSyn-DIO-hM3D(Gq)-mCherry (OBIO company, for specific activation) or AAV2/9-hSyn-DIO-hM4D(Gi)-mCherry (OBIO company, for specific inhibition) was injected into the bilateral SC. These viruses carry *LoxP* elements, enabling specific activation or inhibition of GAD2-positive neurons originating from the mSNr, thereby achieving chemogenetic modulation. On day 1, the AAV2/9-vGAT1-Cre-WPRE virus was bilaterally injected into the medial region of SNr in C57BL/6J mice. Additionally, either AAV2/9-hSyn-DIO-hM3D (Gq)-mCherry or AAV-hSyn-DIO-hM4D (Gi)-mCherry was administered bilaterally into SC. From day 19 to day 21, TAA was administered intraperitoneally on a daily basis to establish a model of AHE. On day 22, the mice were acclimated to darkness. On day 23, visual electrophysiological evaluations were performed. CNO (20 mg/kg) was administered via intraperitoneal injection 30 min prior to the electrophysiological assessments. The samples were divided into four groups: Gi + Saline, Gi + Saline + TAA, Gi + CNO, and Gi + CNO + TAA. The SC coordinates are as follows: 1 mm lateral to the bregma, 3.3 mm posterior to the bregma, and 2.1 mm deep, denoted as X = ± 1 mm; Y = −3.3 mm; Z = −2.1 mm.

#### Isolation of mouse retinal tissue

Following euthanasia, the cheek skin of the mouse was stretched to expose the eyeball outside the orbit. The eyeball was carefully extracted using either straight or curved forceps and placed in a dish containing PBS buffer to rinse off blood. Under a microscope, any muscle tissue adhering to the eyeball was meticulously removed. The eyeball was then incised along the corneal edge, the choroid and sclera were separated, and the lens was carefully removed. Special attention was given to ensure the delicate separation of the transparent retinal tissue during the procedure.

#### Western blot analysis

Retinal tissues were homogenized in modified RIPA lysis buffer to extract proteins, followed by the addition of Halt protease inhibitor. Proteins were separated via 12.5% SDS-PAGE, transferred to membranes, and blocked. Primary antibodies, rabbit anti-GAD65 + 67 (1:500, Cat. #ab183999, Abcam, USA) or mouse anti-β-ACTIN (loading control, 1:5000, Cat. #AC004, Abclonal, China), were incubated overnight at 4°C. Secondary antibodies, HRP-conjugated goat anti-mouse (1:5000, Cat. #A21010, Abbkine, China) and HRP-conjugated goat anti-rabbit (1:5000, Cat. #A21020, Abbkine, China), were incubated at 37°C for 2 h. Chemiluminescence imaging was performed with Fusion FX EDGE, and grayscale values were analyzed using ImageJ. The uncropped WB images can be found in Data S1 in the Supplemental PDF.

### Quantification and statistical analysis

All data are expressed as mean ± standard deviation, with error bars indicating the standard deviation. Statistical analyses were conducted using SPSS 26.0 and GraphPad Prism 8.02. For normally distributed continuous data, paired t-tests or independent sample t-tests were applied to compare two groups. For categorical data, Fisher’s exact test or Pearson’s chi-square test was utilized. Comparisons among three or more groups were performed using one-way ANOVA or two-way ANOVA, followed by Tukey or LSD post hoc tests based on specific conditions. *p*-values less than 0.05 were considered statistically significant.

This table details which specific experiments and viral tracing procedures were performed on each mouse strain (C57BL/6J, c-Fos-CreERT2, GAD2-Cre, GFP-Mito, and GAD2-Mito-GFP) used in the study.

## Supplemental information

Document S1. Figure S1 and Data S1

Table S1. Experimental allocation of transgenic mouse strains across methodological procedures
