# Supplementary material for: Activation of SNr GABA neurons drives liver-brain-eye axis dysfunction in hepatic encephalopathy
Source: iScience. 2026 Jan 16;29(2):114720. doi: 10.1016/j.isci.2026.114720 (PMC12907090; doi:10.1016/j.isci.2026.114720)

iScience, Volume *29*

Supplemental information

Activation of SNr GABA neurons drives liver-brain-eye axis dysfunction in hepatic encephalopathy

Kenan Li, Zhenhua Wang, Shaoheng Li, Feifei Wu, Yunhu Bai, Shujiao Li, Changlei Zhu, Ziwei Ni, Shuai Zhang, Yousheng Wu, Fei Tian, Nannan Liu, Tao Chen, Cailian Ruan, Zuoming Zhang, Yanling Yang, and Yayun Wang

Supplementary Figure1

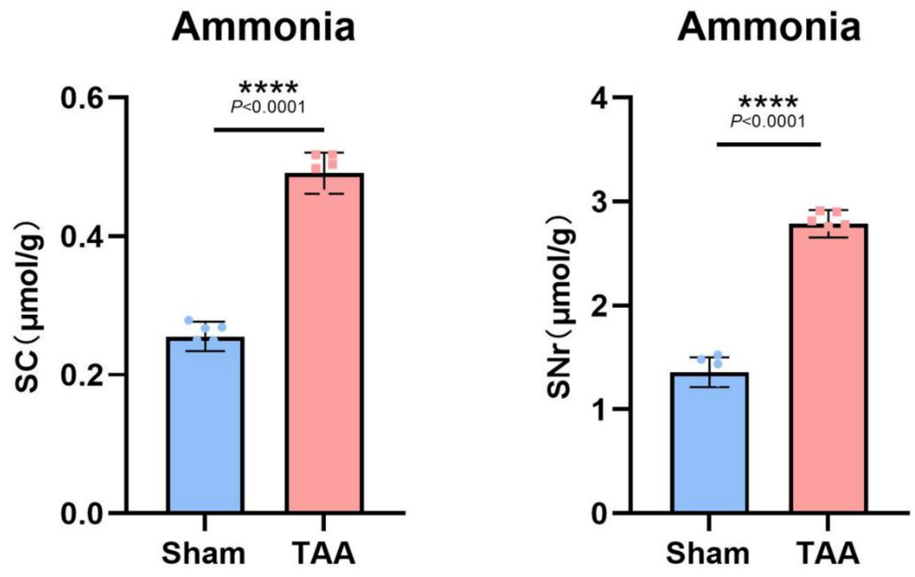

Supplementary Figure1: Effects of TAA treatment on ammonia levels in the SC and SNr regions.

ELISA assays were performed to measure the ammonia concentration in the SC (left) and SNr (right) regions of the brain in Sham and TAA mice on day 4 after TAA injection (n = 6 per group).

All data are presented as mean ± standard deviation. **** $P < 0.0001$. Two tailed, unpaired, Student's t-test for all. data.

Data S1: Uncropped WB Images

Figure 7J
The size of molecular markers: GAPDH (37kDa)
The size of the bands: 37kDa
The name of the experimental groups/treatments: Control vs. AHE

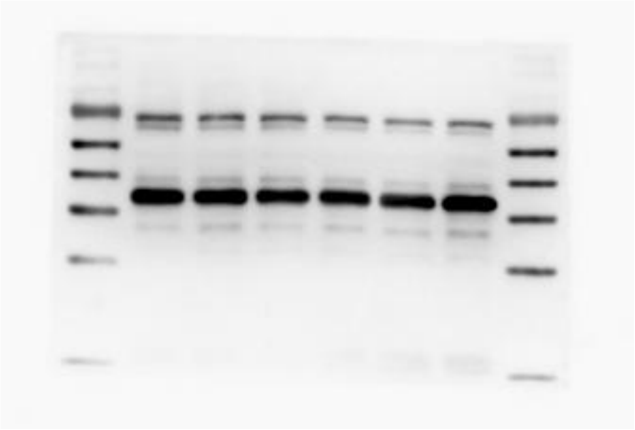

Figure 7J
The size of molecular markers: GAD65/67 (65kDa)
The size of the bands: 65kDa
The name of the experimental groups/treatments: Control vs. AHE

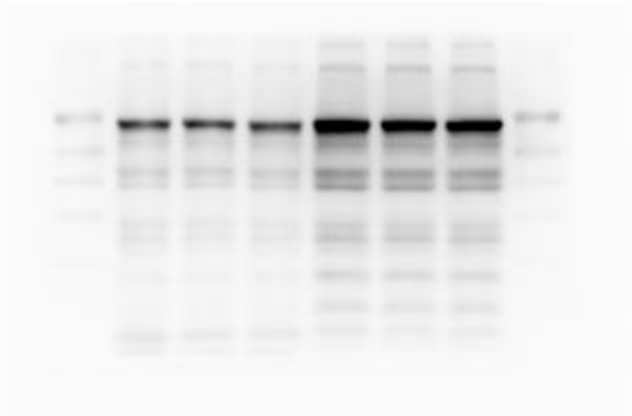

Figure 7L
The size of molecular markers: GAPDH (37kDa)
The size of the bands: 37kDa
The name of the experimental groups/treatments: Sham vs. CHE

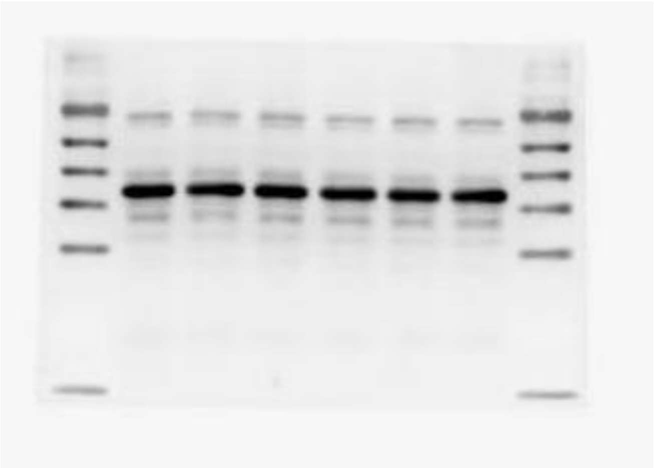

Figure 7L
The size of molecular markers: GAD65/67 (65kDa)
The size of the bands: 65kDa
The name of the experimental groups/treatments: Sham vs. CHE

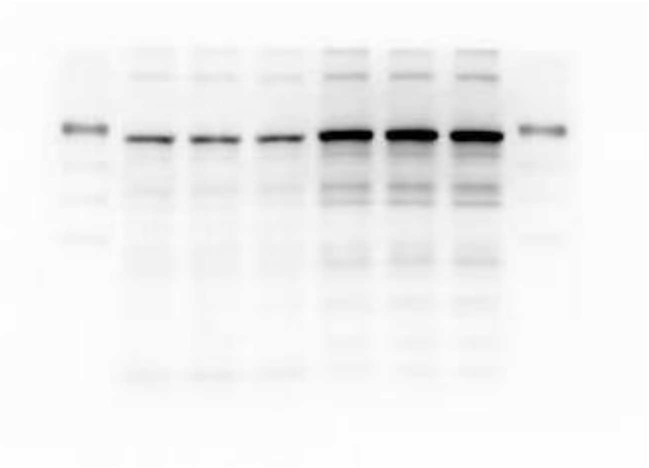

Figure 7N
The size of molecular markers: GAPDH (37kDa)
The size of the bands: 37kDa
The name of the experimental groups/treatments: Control vs. AHE+Saline vs. AHE+CNO

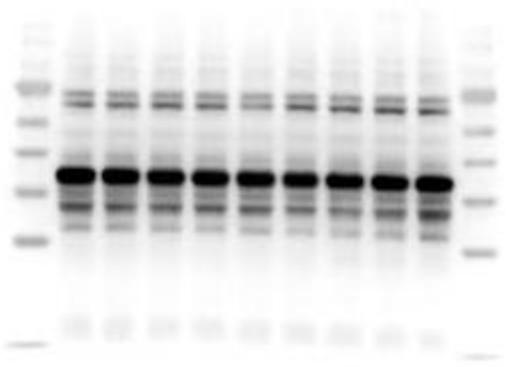

Figure 7N

The size of molecular markers: GAD65/67 (65kDa)

The size of the bands: 65kDa

The name of the experimental groups/treatments: Control vs. AHE+Saline vs. AHE+CNO

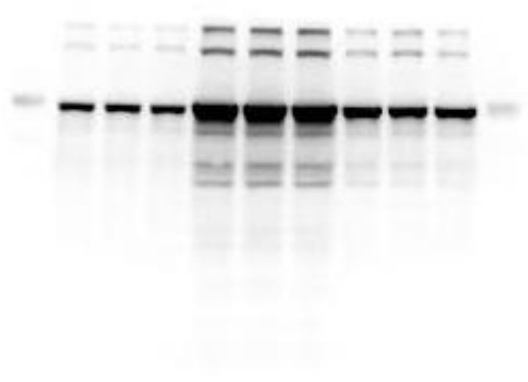

Supplement: Document S1. Figure S1 and Data S1 [file mmc1.pdf]
